# Hypoxia-injured pulmonary vascular targeting and microenvironment-responsive nanoparticle to treat hypoxic pulmonary hypertension

**DOI:** 10.1186/s12951-026-04680-z

**Published:** 2026-07-13

**Authors:** Hongyan Wu, Ruilin Zhou, Wenting Hui, Sai Qiao, Luoqi Zhang, Qifeng Ji, Miao Liu, Bangle Zhang, Shaobo Bai, Daozhou Liu, Siyuan Zhou

**Affiliations:** 1 Department of Pharmaceutics, School of Pharmacy, Air Force Medical University, Xi’an, 710032 China; 2 Department of Cardiology, Department of Internal Medicine, Peking Union Medical College Hospital, Chinese Academy of Medical Science and Peking Union Medical College, Beijing, 100730 China; 3 Department of Pharmacy, The 942th Hospital of Joint Logistic Support Force of PLA, Yinchuan, 750001 China

**Keywords:** Hypoxic pulmonary hypertension, siHIF-1α, Fucoidan, SS31, Pulmonary vascular remodeling

## Abstract

Hypoxic pulmonary hypertension (HPH) is characterized by sustained pulmonary vasoconstriction and vascular remodeling caused by chronic hypoxia. The vicious cycle between hypoxia-inducible factor-1α (HIF-1α) activation and reactive oxygen species (ROS) burst release plays a central role in disease progression. P-selectin is overexpressed on hypoxia-injured pulmonary vascular endothelial cells and pulmonary smooth muscle cells. Fucoidan (Fuco) is a high-affinity ligand for P-selectin. Based on this, a hypoxia-injured pulmonary vascular targeting drug delivery system siHIF-1α/PSS31@Fuco (HPF) was developed. HPF was able to target for hypoxia-injured pulmonary vascular endothelial cells and pulmonary smooth muscle cells via the P-selectin, then HPF was taken up by those cells via caveolin-1-dependent endocytosis. Subsequently, the ROS-triggered depolymerization of PSS31 ensured efficient cytosolic release of siHIF-1α and SS31 to efficiently inhibit the expression of HIF-1α and the production of ROS, respectively. In HPH rats, HPF alleviated pulmonary vascular remodeling and improved the hemodynamics and right ventricular function by breaking the vicious cycle between HIF-1α activation and ROS production. Therefore, HPF is a promising nanotherapeutic strategy for HPH.

**Graphical Abstract:**

**Figure:**
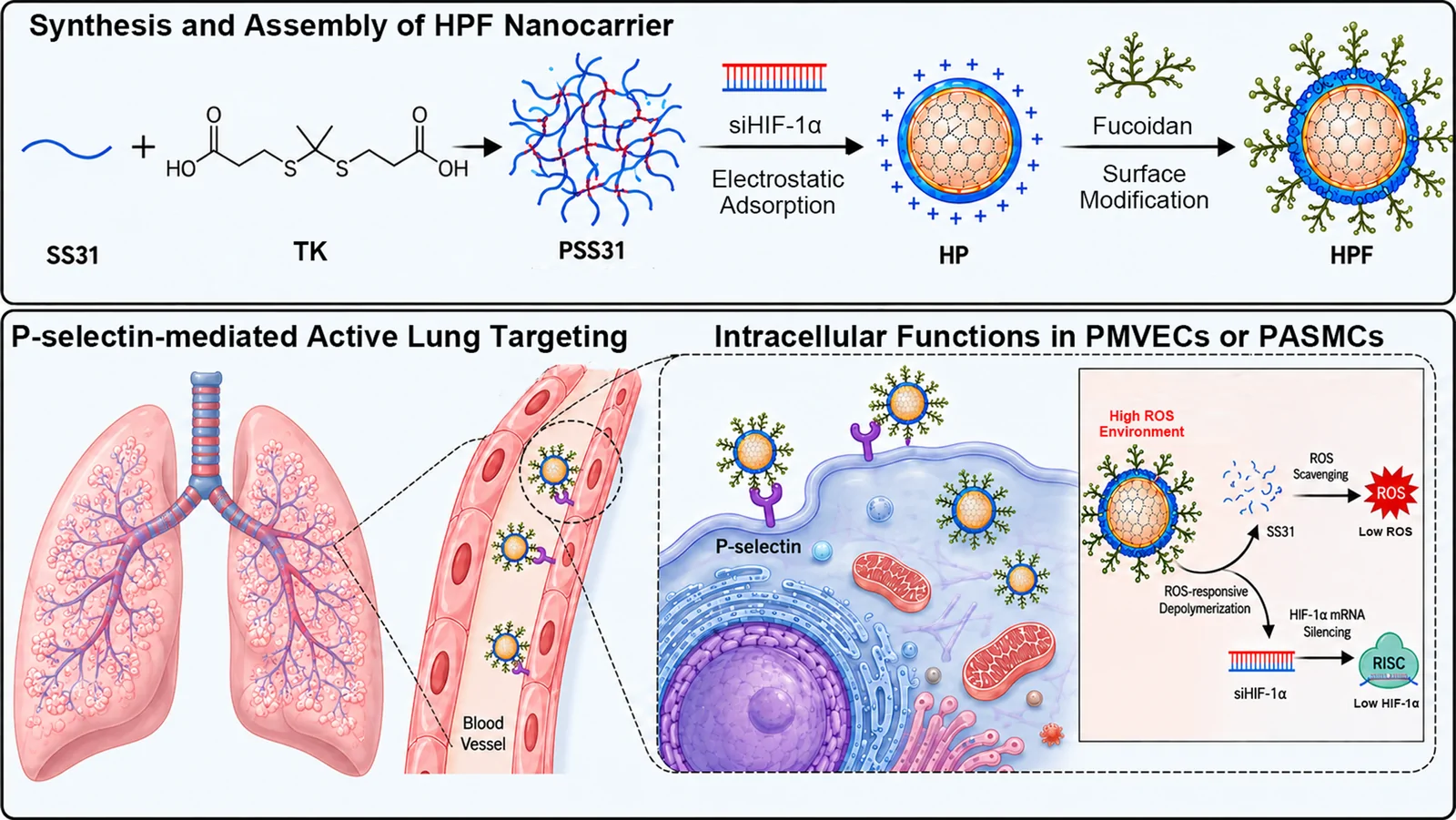

**Supplementary Information:**

The online version contains supplementary material available at https://doi.org/10.1186/s12951-026-04680-z.

## Introduction

Hypoxic pulmonary hypertension (HPH) is a progressive and life-threatening cardiopulmonary disorder classified as WHO Group 3 pulmonary hypertension. It is characterized by sustained pulmonary arterial hypertension resulting from chronic hypoxia [1, 2]. The pathological hallmarks of HPH include sustained pulmonary vasoconstriction and arterial remodeling, leading to an elevation of right ventricular afterload and right heart failure [3, 4]. Current standard treatments such as oxygen supplementation and non-specific vasodilators provide only limited symptomatic relief. They fail to inhibit vascular remodeling and are associated with considerable adverse effects [5–8]. To date, a 5-year mortality rate of HPH exceeds 60% [9].

Pulmonary vascular remodeling serves as the critical structural basis for the progression and irreversibility of HPH [10]. Under chronic hypoxic condition, pulmonary microvascular endothelial cells (PMVECs) exhibit dysfunction, leading to disruption of intercellular junctions of PMVECs and impairment of the vascular barrier function. Concurrently, endothelial injury shifts the balance of vasoactive mediators, with reduced levels of vasodilators (e.g., nitric oxide, NO) and increased expression of vasoconstrictors (e.g., endothelin-1, ET-1) [11, 12]. Notably, the pathological alterations in cells under HPH condition are not isolated events. ET-1 secreted by PMVECs acts in a paracrine manner on the endothelin A receptor (ETA) expressed on pulmonary arterial smooth muscle cells (PASMCs), thereby promoting their contraction, hyperproliferation, and resistance to apoptosis, which in turn accelerates disease progression [13].

Hypoxia-inducible factor-1α (HIF-1α), a pivotal regulator of hypoxic responses, is activated under hypoxic condition due to suppressed prolyl hydroxylase activity. As a critical transcription factor, HIF-1α transcriptionally activates multiple pathogenic genes associated with HPH, aggravating vasoconstriction and vascular cell proliferation [14, 15]. Additionally, HIF-1α upregulates NADPH oxidase and disturbs mitochondrial function, resulting in the massive production of reactive oxygen species (ROS) [16]. As a second messenger, ROS not only induces vasoconstriction and proliferation of pulmonary arterial smooth muscle cells but also stabilizes HIF-1α, thus setting up a pathological positive-feedback loop [17, 18]. Therefore, inhibiting the expression of HIF-1α and the excessive production of ROS hold great therapeutic promise for HPH.

The small interfering RNA against HIF-1α (siHIF-1α) offers a specific approach to treat HPH. Although several siRNA therapeutics have received FDA approval [19], the efficient and targeting delivery of siHIF-1α to the pulmonary vasculature remains a challenge. This challenge arises from the inherent physicochemical properties of siRNA and complex in vivo biological barriers, including rapid degradation by plasma nucleases, non-specific binding with serum proteins, and inefficient cellular uptake. Notably, lysosomal escape, the critical step for siRNA delivery, also remains inefficient even when siRNA is taken up by the targeting cells [20, 21].

It has been documented that the expression of P-selectin and caveolin-1 are significantly upregulated in both PMVECs and PASMCs in patients with pulmonary arterial hypertension (PAH) [22]. P-selectin is a type I transmembrane glycoprotein. As a cell adhesion molecule, it is rapidly activated and expressed on the cell surface upon injurious stimuli, serving as a key mediator of immune cell adhesion and migration to inflammatory sites. Caveolin-1 is the main structural protein of caveolae, which can regulate the molecules enriched in specific concave areas of the cell membrane and participate in the endocytosis process of extracellular substances. siRNA delivery system can be guided to the Golgi body or endoplasmic reticulum through the endocytosis pathway mediated by caveolin-1, thus avoiding fusion with lysosomes. This effectively reduces the phagocytosis and degradation of siRNA by lysosomes, ultimately improving the silence efficiency of the siRNA delivery system [23, 24]. In addition, fucoidan (Fuco), a type of algal polysaccharide, is a highly affinity ligand for P-selectin. Taking advantage of the overexpression of P-selectin and caveolin-1 in pulmonary vessel cells of HPH, Fuco modified drug delivery systems can targeting the diseased vascular cells of HPH via the P-selectin, then it is internalized into diseased vascular cells through caveolin-1-dependent endocytosis, effectively avoiding lysosomal degradation of drug [25].

**Scheme 1:**
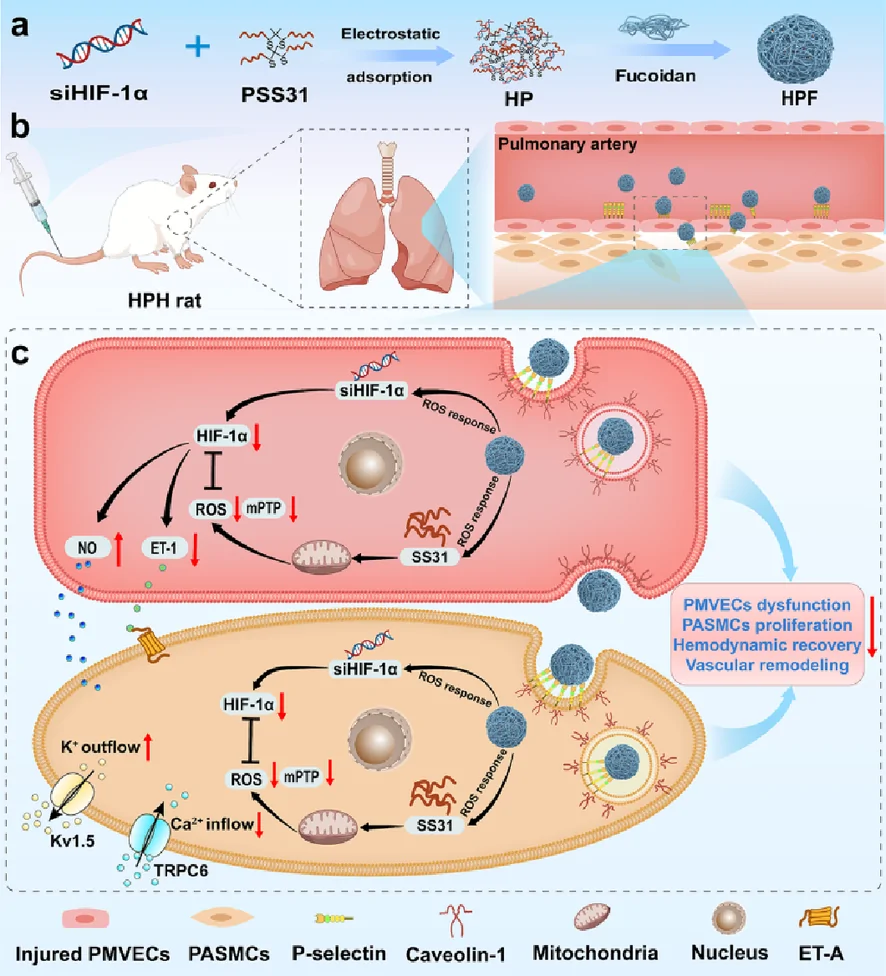
Schematic illustration of the preparation and therapeutic effect of HPF. (**a**) Schematic illustration of the HPF fabrication process. (**b**) Tail vein injection of HPF can efficiently targeting the pulmonary vasculature in HPH rats. (**c**) Treatment with HPF effectively disrupted the vicious cycle between activation of HIF-1α and production of ROS, ameliorated mitochondrial dysfunction, and ultimately improved hemodynamics and vascular remodeling in HPH rats by alleviating PMVECs dysfunction and suppressing PASMCs hyperproliferation

 The mitochondrial targeting antioxidant peptide SS31 shows positively charged under physiological conditions. In addition, SS31 can inhibit mitochondrial cardiolipin oxidation, thereby reducing the generation of mitochondrial ROS [26, 27]. However, the weak cationic character of SS31 hinders effective complexation with negatively charged siHIF-1α [28]. In this study, the ROS-responsive polymer carrier PSS31 was synthesized by polymerizing SS31 with thioketal (TK), a ROS-cleavable linkage containing two carboxyl groups. The siHIF-1α/PSS31 complex (HP) was formed by loading siHIF-1α onto PSS31 via electrostatic adsorption. Subsequently, HPF was prepared to targeting the injured lung vascular cells by modifying HP with Fuco. The therapeutic effects of HPF on HPH in rat were explored in vitro and *in viv*o, providing a new strategy for the targeting therapy for HPH (Scheme 1).

## Results and discussion

### Characterization of HPF

**Fig. 1:**
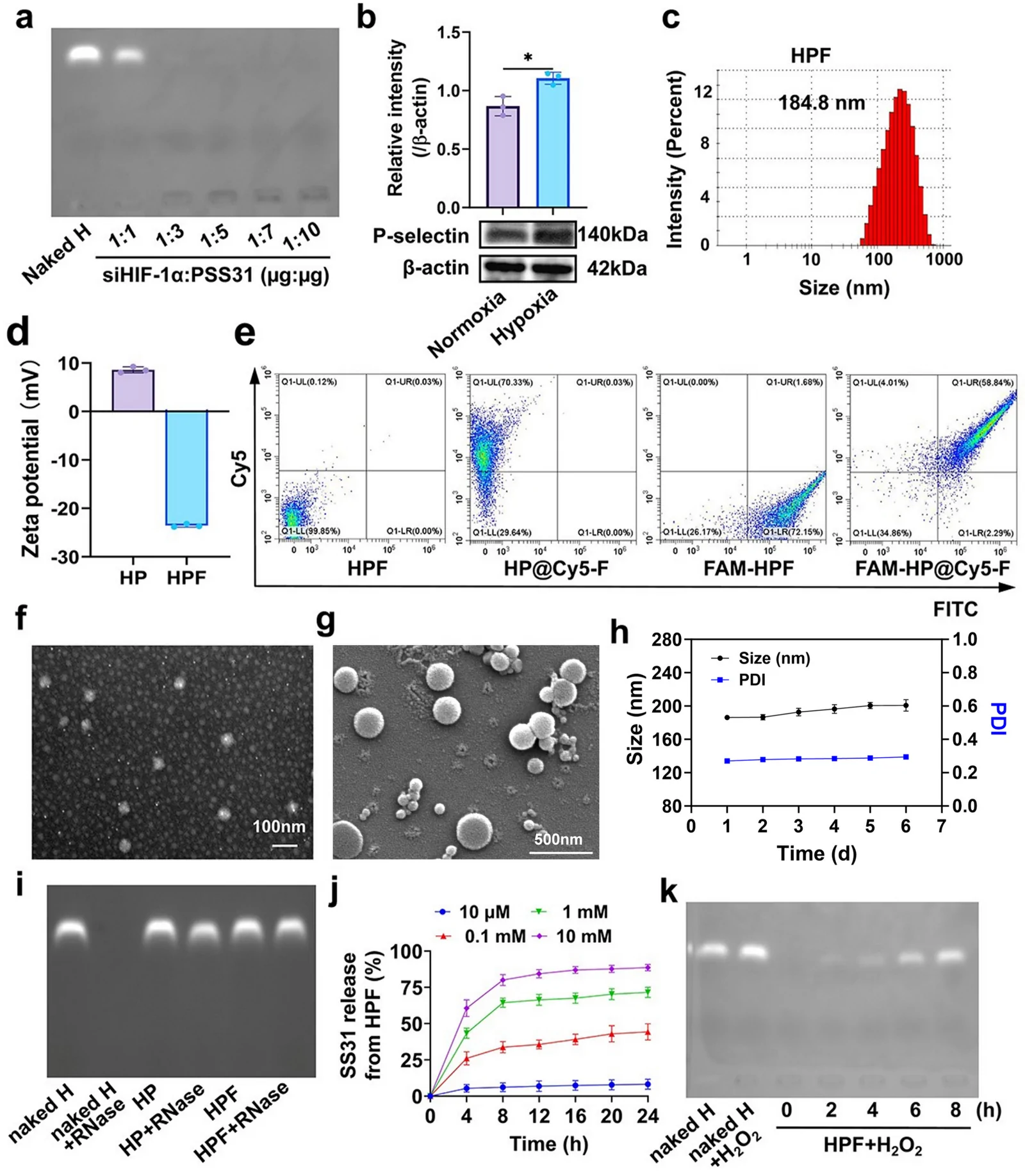
Preparation and characterization of HPF. **(a**) Screening of the optimal feeding ratio of siHIF-1α to PSS31 by agarose gel electrophoresis. (**b**) Representative western blot and statistical analysis of P-selectin protein in lung tissues of HPH Rats. (**c**) Particle size of HPF. (**d**) Zeta-potential of HP and HPF. (**e**) The adsorption of FAM-siHIF-1α with Cy5-Fuco was analyzed by flow cytometry. (**f**) Transmission electron microscopy image of HPF. (**g**) Scanning electron microscopy image of HPF. (**h**) Stability of HPF within 6 days in PBS solution. (**i**) Stability of siHIF-1α in RNase-containing solution. (**j**) SS31 release from HPF in H_2_O_2_-containing buffer. (**k**) siHIF-1α release from HPF in H_2_O_2_-containing buffer. Naked H presents the naked siHIF-1α. Data were presented as mean ± SD, *n* = 3. ^*^P < 0.05. Statistical significance was calculated via unpaired two-tailed Student’s t-test

Western blot analysis was used to screen the siRNA sequences of HIF-1α under hypoxic condition. Among these, siHIF-1α−2310 demonstrated the best potential in silencing HIF-1α expression in PMVECs (Fig.S1), and its detailed sequences are provided in Tab.S1. Then, SS31 was polymerized to form a polymeric carrier PSS31 using a ROS-responsive thioketal (TK) containing carboxyl groups (Fig.S2). The theoretical molecular weight of PSS31 was calculated to be 3208.15. Accordingly, the predicted m/z values were 3211.174, 3212.182, and 3213.190 for the [M + 3 H]³⁺, [M + 4 H]⁴⁺, and [M + 5 H]⁵⁺, respectively. Time-of-flight mass spectrometry (TOF MS) results closely matched these predictions ([M + 3 H]³⁺: 3211.647; [M + 4 H]⁴⁺: 3212.624; [M + 5 H]⁵⁺: 3213.645), confirming the successful synthesis of PSS31 (Fig.S3). As revealed by the gel retardation assay, complete complexation of siHIF-1α with PSS31 occurred at a mass ratio of 1:5 (siHIF-1α:PSS31), leading to the formation of stable siHIF-1α/PSS31 complex (HP) (Fig. 1a).

P-selectin was markedly upregulated in rat lung tissues following 4-week hypoxia exposure (Fig. 1b). Fuco, a high affinity P-selectin ligand, confers a strong negative charge under physiological conditions [29]. Thus, the lung targeting siHIF-1α delivery system HPF was constructed by adsorbing Fuco onto the positively charged HP surface. HP showed an average hydrodynamic diameter of 161.5 nm and a zeta potential of +8.05 mV. After Fuco coating, the diameter increased to 184.8 nm, with a corresponding marked reversal of the zeta potential to −23.8 mV (Fig. 1c, d, Fig.S4). These shifts in size and surface charge strongly indicated successful coating of HP with Fuco. Dual-fluorescence flow cytometry analysis showed the dual-labeled HPF (FAM-HP@Cy5-F) were overwhelmingly detected in quadrant 1 (Fig. 1e), indicating the co-localization of siHIF-1α and Fuco within HPF. This further confirmed the efficient incorporation of HP and Fuco.

Transmission electron microscopy (TEM) imaging revealed that HPF adopted a uniform spherical morphology with a narrow size distribution (Fig. 1f). Scanning electron microscopy (SEM) further confirmed that the HPF possessed regular spherical structures with good dispersibility (Fig. 1g). The HPF exhibited colloidal stability in phosphate-buffered saline (PBS), maintaining their structural integrity over 6 days without significant aggregation (Fig. 1h). Furthermore, the particle size and PDI of HPF remained stable in DMEM containing 10% fetal bovine serum (FBS) over 72 h incubation, confirming their stability under simulated physiological condition (Fig.S5). Next, the capacity of HPF to protect siHIF‑1α against enzymatic degradation was evaluated. While naked siHIF‑1α was completely degraded after 2 h incubation with RNase, the siRNA encapsulated within HPF remained intact (Fig. 1i). Then, liquid chromatography-mass spectrometry (LC-MS) was used to monitor SS31 peptide release. The results indicated that HPF released SS31 in H₂O₂ concentration-dependent manner (Fig. 1j). Similarly, gel electrophoresis analysis showed a ROS-dependent release of siHIF‑1α from HPF (Fig. 1k). These data confirmed the ROS-responsive drug release capability of HPF, enabling the synchronized release of both siHIF‑1α and SS31 in oxidative stress microenvironments.

Hemocompatibility analysis showed hemolysis rates below 5% across all tested HPF concentrations (Fig.S6). Subsequently, MTT assay revealed high cell viability in both PMVECs and PASMCs across all tested HPF concentrations (Fig.S7). These results established the in vitro biosafety of HPF.

### HPF accumulated in PMVECs and PASMCs *via* P-selectin mediated targeting and caveolin-1 mediated endocytosis

**Fig. 2:**
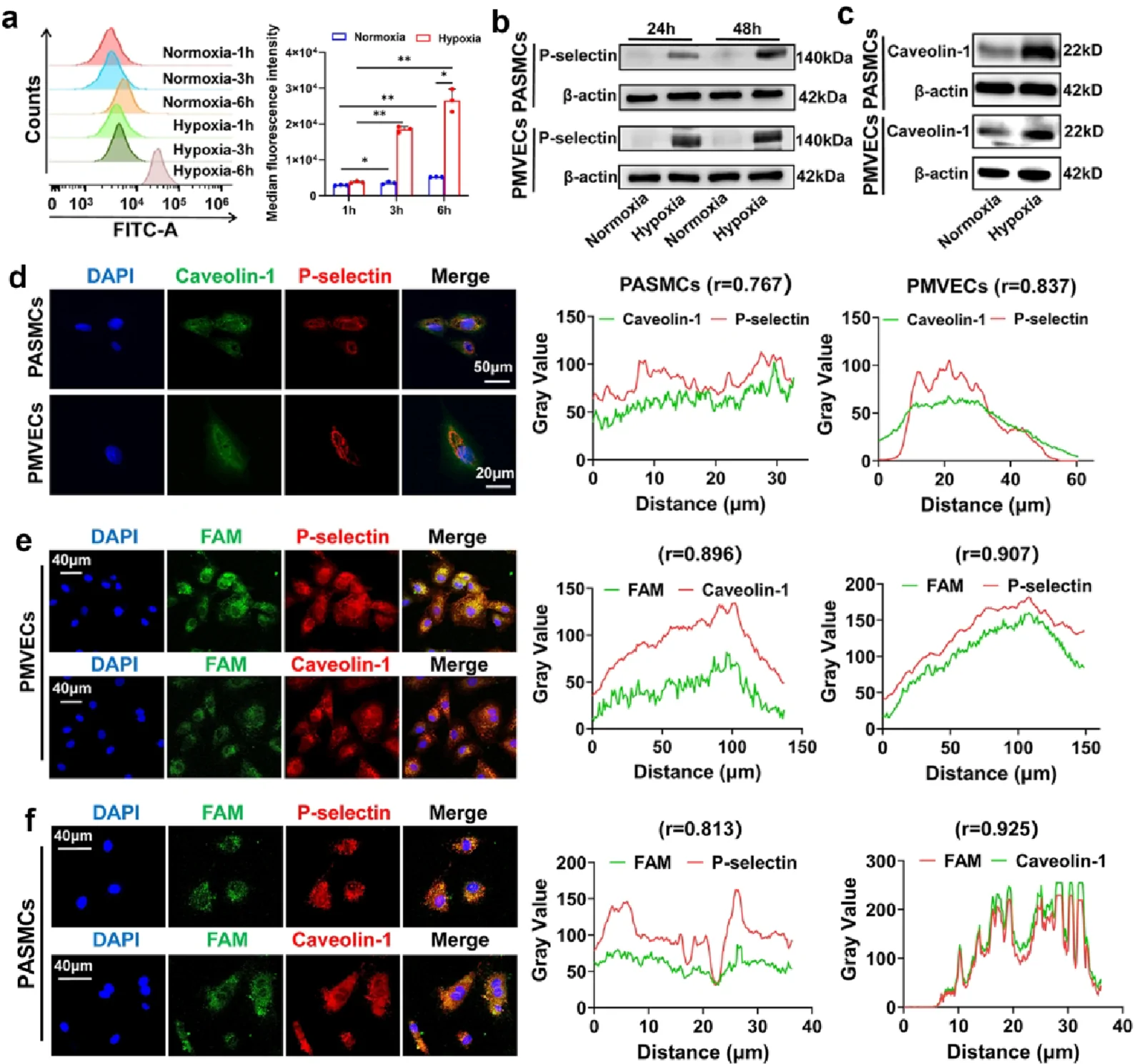
HPF accumulated in PMVECs and PASMCs *via* P-selectin mediated targeting and caveolin-1 mediated endocytosis. (**a**) Cellular uptake of HPF in normoxic or hypoxic environment in PMVECs was determined using flow cytometry. (**b**) Western blot analysis of P-selectin expression in PMVECs and PASMCs under normoxic or hypoxic condition for 24–48 h. (**c**) Western blot analysis of caveolin-1 protein expression in PMVECs and PASMCs under normoxic or hypoxic condition. (**d**) Immunofluorescence staining for the colocalization of P-selectin and caveolin-1 in hypoxia-injured PMVECs and PASMCs (red: P-selectin; green: caveolin-1; blue: DAPI). (**e**) Immunofluorescence staining for the colocalization of FAM-HPF with P-selectin or caveolin-1 in hypoxia-injured PMVECs after 1 h incubation (red: P-selectin and caveolin-1; green: FAM-labelled HPF; blue: DAPI). **(f**) Immunofluorescence staining for the colocalization of FAM-HPF with P-selectin or caveolin-1 in hypoxia-injured PASMCs after 1 h incubation (red: P-selectin and caveolin-1; green: FAM-labelled HPF; blue: DAPI). Data were presented as mean ± SD, *n* = 3. ^**^P < 0.01. Statistical significance was calculated *via* two-tailed Student’s t test

At first, the primary PMVECs (CD31^+^) and PASMCs (α-SMA^+^) were isolated and purified (Fig.S8). Then, the isolated PMVECs and PASMCs were separately seeded into different cell culture plates, and the accumulation of HPF in PMVECs and PASMCs was detected by flow cytometry. The results revealed that the accumulation of HPF in hypoxic PMVECs and PASMCs was higher than that of HP (Fig. 2a and Fig.S9), indicating that Fuco enhanced targeting capability of HPF to hypoxic PMVECs and PASMCs, and P-selectin was involved in the accumulation of HPF in hypoxic PMVECs and PASMCs.

Western blot results indicated that a remarkable upregulation of caveolin-1 in lung tissues of HPH rats (Fig.S10). Besides, the expression of P-selectin and caveolin-1 were significantly elevated in hypoxia-injured PMVECs and PASMCs (Fig. 2b, c, Fig.S11). Notably, immunofluorescence staining demonstrated substantial intracellular colocalization of P-selectin and caveolin-1 (Fig. 2d), suggesting their potential cooperation in mediating HPF cellular uptake by hypoxia-injured PMVECs and PASMCs. To test this hypothesis, the intracellular trafficking of HPF in hypoxia-injured PMVECs and PASMCs was investigated. After hypoxia-injured PMVECs and PASMCs were treated with FAM-labelled HPF (FAM-HPF), a clear colocalization of the FAM signal with both P-selectin and caveolin-1 was observed (Fig. 2e, f). Finally, the uptake mechanism of HPF in PMVECs and PASMCs was investigated. Colchicine showed the strongest inhibitory effect on the uptake of HPF by normal PMVECs and PASMCs, whereas MβCD exerted the strongest inhibition on the uptake of HPF by hypoxia-injured PMVECs and PASMCs (Fig.S12, S13). These results suggested that the uptake of HPF by normal PMVECs and PASMCs was mainly mediated via macropinocytosis. However, the uptake of HPF by hypoxia-injured PMVECs and PASMCs was predominantly mediated by caveolin-1-dependent endocytosis. Collectively, these findings demonstrated that HPF accumulated in PMVECs and PASMCs *via* P-selectin targeting and caveolin-1 mediated endocytosis.

### The lysosomal escape, trans-endothelial transport and macrophage evasion of HPF

**Fig. 3:**
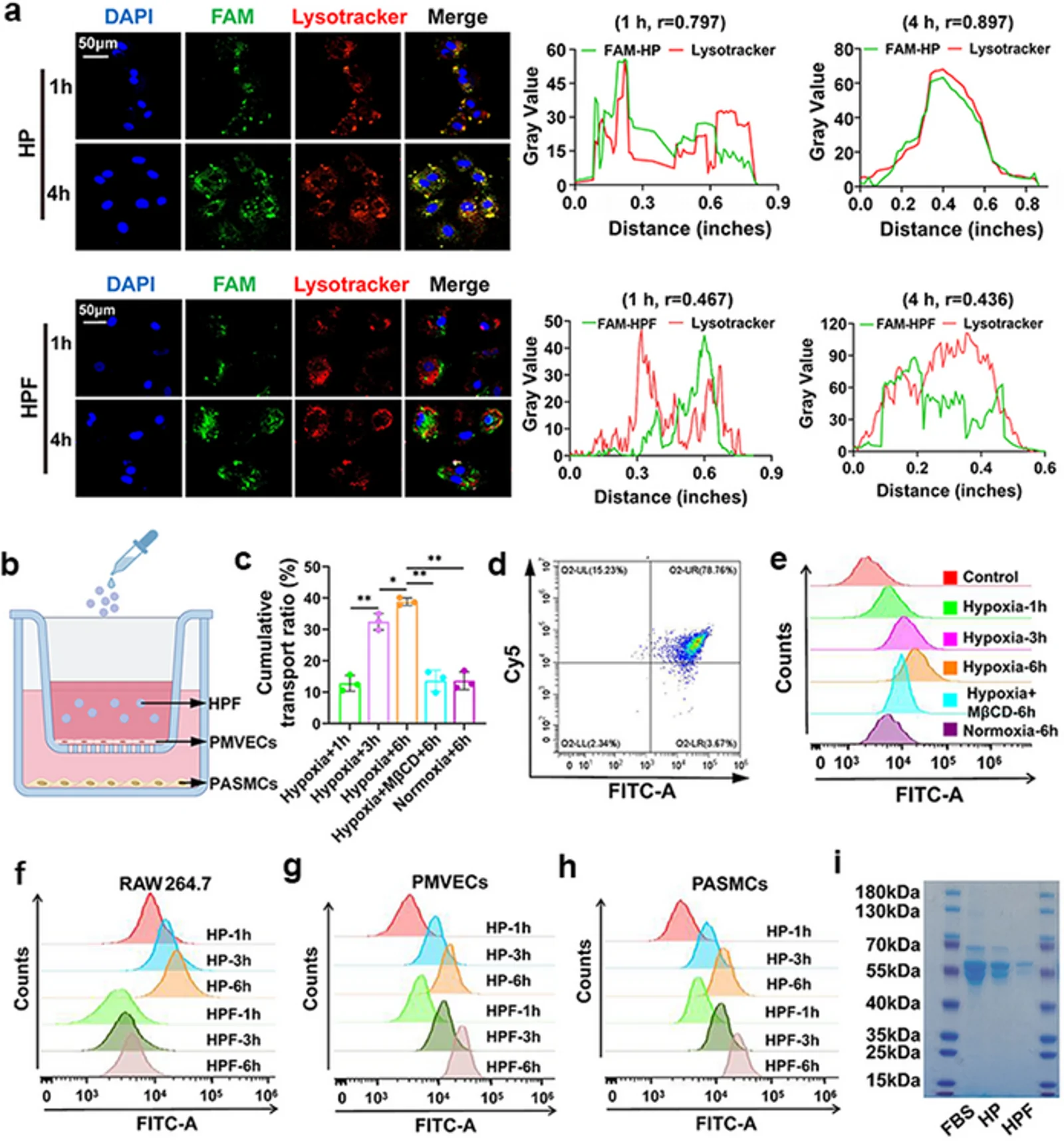
The lysosomal escape, trans-endothelial transport and macrophage evasion of HPF. (**a)** Immunofluorescence staining for the colocalization of FAM-HP and FAM-HPF with lysosome in hypoxia-injured PMVECs after 1–4 h incubation (red: lysosome labeled with Lysotracker; green: FAM-labelled HP or FAM-labelled HPF; blue: DAPI). (**b**) Schematic diagram of the in vitro trans-endothelial transport model. (**c**) The cumulative transport rate of HPF across in vitro trans-endothelial barrier. (**d**) Flow cytometric analysis of HPF after penetrating through endothelial barrier in vitro. (**e**) Accumulation of FAM-HPF in PASMCs after penetrating through endothelial barrier detected by flow cytometry. **(f-h**) Representative flow cytometry images of HP and HPF uptake by RAW264.7 cells, PMVECs and PASMCs in the co-culture system under hypoxic condition. (**i)** SDS-PAGE image of serum protein adsorbed by HP and HPF. Data were presented as mean ± SD, *n* = 3. ^*^P < 0.05, ^**^P < 0.01. Statistical significance was calculated *via* one-way ANOVA with Tukey’s test. ns: no significant difference

A pivotal challenge in RNAi therapy is how to avoid the degradation of siRNA in lysosome [30]. Confocal microscopy revealed that HP presented sustained colocalization with lysosomes, indicating that most HP was entrapped by lysosomes. However, HPF showed minimal colocalization of siHIF-1α with lysosomal at both 1 h and 4 h post-internalization (Fig. 3a), suggesting that HPF could avoid the destruction of lysosomes.

To evaluate the trans-endothelial transport capacity of HPF, an in vitro endothelial barrier model was established using a transwell co-culture system with PMVECs and PASMCs (Fig. 3b). A trans-endothelial electrical resistance (TEER) value of approximately 250 Ω·cm² confirmed the formation of an intact and tight endothelial monolayer [31, 32]. The addition of double labelled HPF (FAM-HP@Cy5-F) did not significantly alter the TEER value within 6 h (Fig.S14), demonstrating that the HPF did not compromise endothelial barrier integrity. The cumulative transport rate of HPF increased progressively over time (Fig. 3c), underscoring its efficient and sustained endothelial barrier-penetrating capability. Flow cytometric analysis showed that the FAM-HP@Cy5-F signal from lower chamber medium was predominantly located in the quadrant 1 (Fig. 3d), confirming that HPF maintained its structural integrity during trans-endothelial transport. Flow cytometry and confocal microscopy analysis of PASMCs in the lower chamber revealed that hypoxia-injured PASMCs exhibited significantly higher fluorescence intensity compared to normal PASMCs or hypoxia-injured PASMCs pretreated with MβCD (Fig. 3e, Fig.S15,16), indicating after penetrating through endothelial barrier, HPF could still be taken up by hypoxia-injured PASMCs through caveolin-1 mediated endocytosis, and more amount of HPF was taken up by hypoxia-injured PASMCs than normal PASMCs.

Non-specific uptake by macrophages represents a major factor that limit the targeting efficiency of siRNA delivery systems [33]. To evaluate the macrophage uptake of HPF, a co-culture system under hypoxia containing RAW264.7 cells, PMVECs, and PASMCs was established. As compared to HP, less amount of HPF was taken up by hypoxia-injured RAW264.7 cells. A larger amount of HPF was taken up by hypoxia-injured PMVECs and PASMCs (Fig. 3f-h, Fig.S17), indicating HPF was preferentially and efficiently internalized by PMVECs and PASMCs. Besides, HPF demonstrated greater stability in size and PDI index in solution containing 10% fetal bovine serum (FBS) compared to HP (Fig.S18). SDS-PAGE analysis further revealed minimal protein adsorption onto HPF (Fig. 3i). These results demonstrated that Fuco on the HPF surface formed strong hydrogen bonds with water molecules, establishing a protective hydration barrier. On the one hand, the hydration barrier reduced the recognition and subsequent internalization by RAW264.7 cells. On the other hand, this barrier effectively reduced serum protein adsorption, thereby enhanced the recognition and subsequent internalization by hypoxia-injured PMVECs and PASMCs.

### HPF ameliorated oxidative stress injury in PMVECs and PASMCs by inhibiting the expression of HIF-1α and the production of ROS

**Fig. 4:**
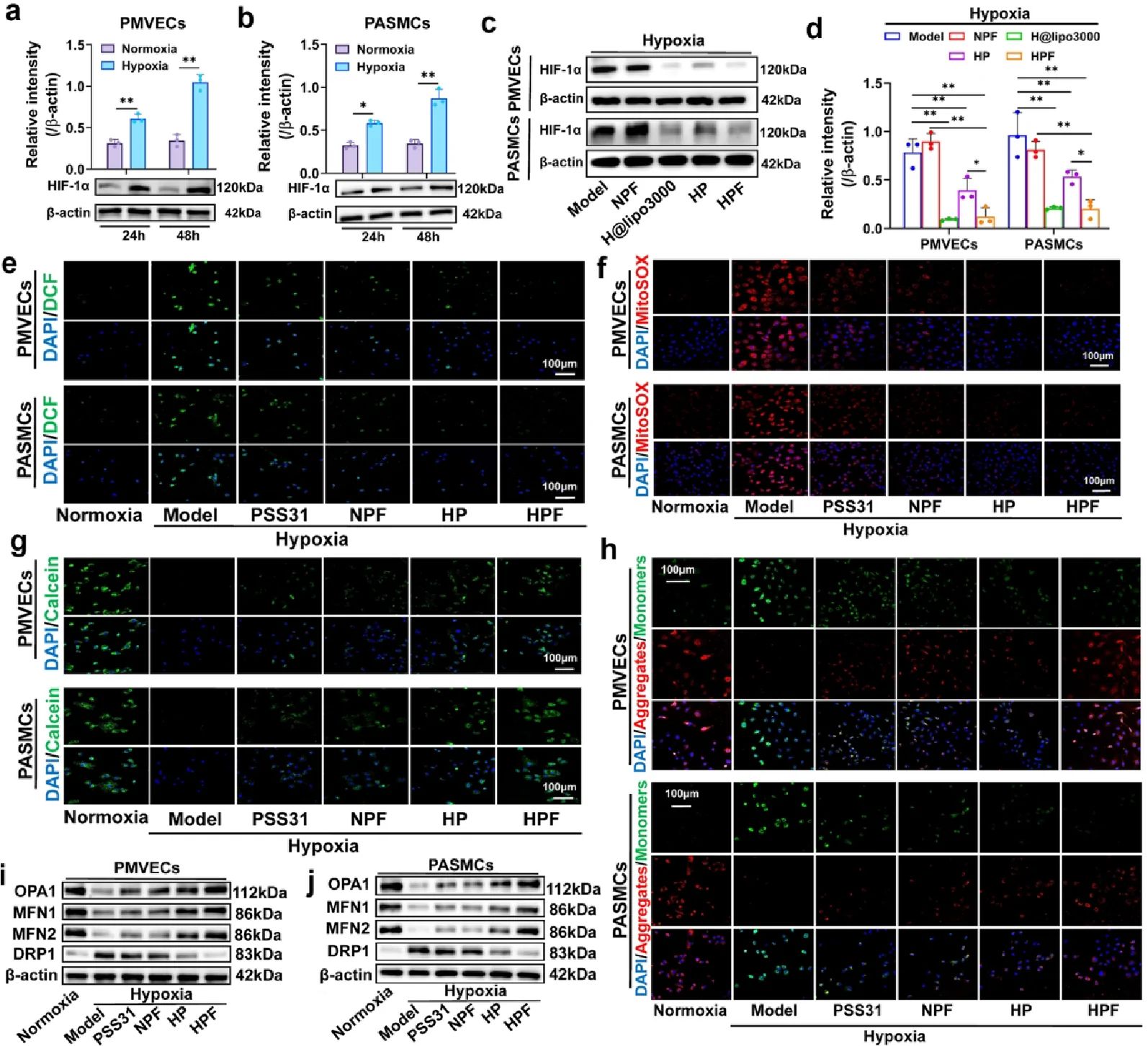
HPF ameliorated oxidative stress injury in PMVECs and PASMCs by inhibiting the expression of HIF-1α and the production of ROS. (**a, b**) Representative immunoblots and statistical analysis of HIF-1α expression in PMVECs and PASMCs under normoxic or hypoxic conditions for 24–48 h. (**c, d)** Representative immunoblots and statistical analysis of HIF-1α expression in PMVECs and PASMCs after treatment with NPF, H@lipofectamine 3000, HP or HPF for 48 h under hypoxic environment. (**e**) LSCM images showing the effects of PSS31, HP, NPF, and HPF on ROS levels in hypoxia-injured PMVECs and PASMCs (green: DCF; blue: DAPI). (**f)** LSCM images showing the effects of PSS31, HP, NPF and HPF on mitochondrial superoxide levels in hypoxia-injured PMVECs and PASMCs (red: MitoSOX; blue: DAPI). (**g**) LSCM images showing the effects of PSS31, NPF, HP and HPF on the opening of mPTP in hypoxia-injured PMVECs or PASMCs (green: Calcein; blue: DAPI). (**h**) LSCM images showing the effects of PSS31, HP, NPF and HPF on the mitochondrial membrane potential in hypoxia-injured PMVECs and PASMCs (green: Monomers; Red: Aggregates; blue: DAPI). (**i, j**) The effect of PSS31, HP, NPF and HPF on the expression of mitochondrial dynamics-related proteins in hypoxia-injured PMVECs and PASMCs. NPF indicates siNC/PSS31@Fuco. Data were presented as mean ± SD, *n* = 3. ^*^P < 0.05, ^**^P < 0.01. Statistical significance was calculated *via* unpaired two-tailed Student’s t-test (**a, b**) or one-way ANOVA with Tukey’s test (**d**)

Western blot results indicated that the HIF-1α protein levels were significantly upregulated in hypoxia-injured PMVECs and PASMCs (Fig. 4a,b). HPF significantly reduced expression of HIF-1α in hypoxia-injured PMVECs and PASMCs as compared with HP (Fig. 4c,d). Immunofluorescence analysis also revealed a time-dependent downregulation of HIF-1α expression following HPF treatment in hypoxia-injured PMVECs (Fig.S19).

Confocal microscopy showed a marked increase in total intracellular ROS levels in hypoxia-injured PMVECs and PASMCs, and HPF exhibited the highest ROS-scavenging efficacy (Fig. 4e and Fig.S20). In addition, mitochondrial superoxide detection revealed that hypoxia significantly enhanced mtROS production, and HPF effectively attenuated mtROS production in hypoxia-injured PMVECs and PASMCs (Fig. 4f and Fig.S21).

To investigate the protective effect of HPF on mitochondrial function, mPTP opening and mitochondrial membrane potential (ΔΨm) were assessed in hypoxia-injured PMVECs and PASMCs. The results showed that mPTP opening was markedly suppressed by HPF (Fig. 4g and Fig.S22) in hypoxia-injured PMVECs and PASMCs. JC-1 staining confirmed that hypoxia significantly reduced the red/green fluorescence ratio, indicating the decrease of ΔΨm in hypoxia-injured PMVECs and PASMCs. HPF substantially increased the red/green fluorescence ratio (Fig. 4h and Fig.S23), demonstrating HPF could restore ΔΨm in hypoxia-injured PMVECs and PASMCs. Western blot analysis revealed that hypoxia disrupted mitochondrial dynamics by downregulating fusion proteins (MFN1, MFN2, OPA1) and upregulating the fission protein DRP1 in hypoxia-injured PMVECs and PASMCs. HPF effectively reversed these aberrant expression patterns, restoring the balance between mitochondrial fission and fusion in hypoxia-injured PMVECs and PASMCs (Fig. 4i, j and Fig.S24). These results indicated that the preservation of mitochondrial function by HPF was mediated, at least in part, through the normalization of mitochondrial dynamics.

Hypoxia induces oxidative stress in PMVECs and PASMCs primarily mediated by mitochondrial dysfunction and the overproduction of mitochondrial ROS (mtROS) [34]. Furthermore, the mtROS burst is known to activate HIF-1α. Then the activated HIF-1α promotes mitochondrial fission and suppresses mitochondrial fusion, thereby sustaining elevated mtROS levels and the expression of HIF-1α [35, 36], which subsequently drives pulmonary vascular remodeling [37]. HPF not only efficiently silenced the abnormally overexpressed HIF-1α, but also attenuated mtROS production *via* its mitochondria-protective effects in hypoxia-injured PMVECs and PASMCs. Therefore, the ameliorative effects of HPF on hypoxia-induced oxidative stress and mitochondrial dysfunction in pulmonary vascular cells were significantly superior to those in PSS31 and NPF groups.

### HPF inhibited the hypoxia-induced migration of PMVECs and improved hypoxia-induced dysfunction of PASMCs

Under hypoxic stress, activation of the HIF-1α/ROS signaling pathway drives pathological PMVECs migration, leading to endothelial detachment and displacement into the smooth muscle layer. Then, the displaced PMVECs promote PASMCs proliferation through paracrine signaling. These events collectively accelerate pulmonary vascular remodeling [38].

**Fig. 5:**
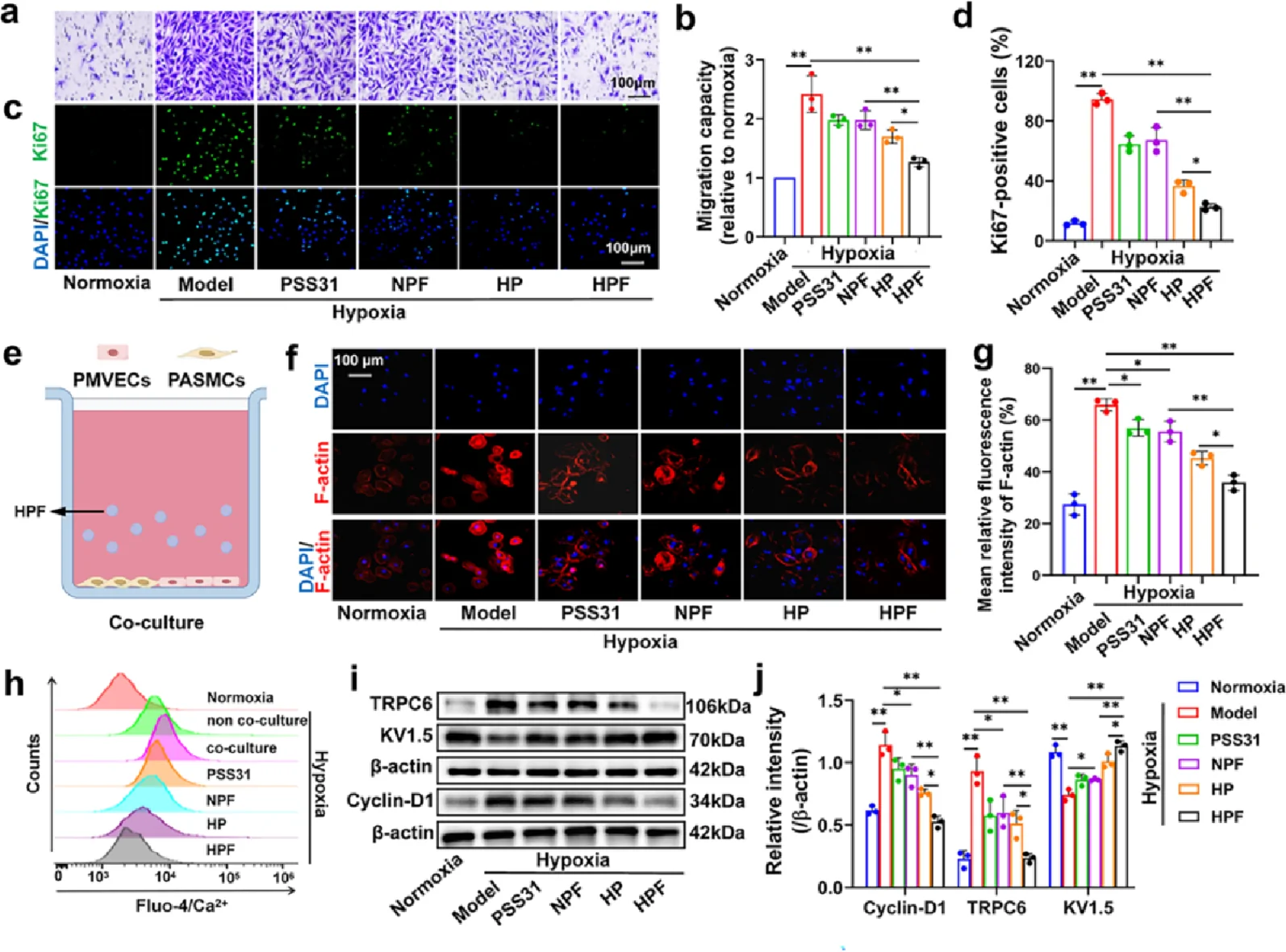
HPF inhibited the hypoxia-induced migration of PMVECs and improved hypoxia-induced dysfunction of PASMCs. (**a, b**) Effects of PSS31, HP, NPF, and HPF on the migration of PMVECs in hypoxia-injured co-culture system. (**c, d**) Effects of PSS31, HP, NPF, and HPF on PASMCs proliferation in hypoxia-injured co-culture system (green: Ki67; blue: DAPI). (**e)** Schematic diagram of the direct co-culture system between PMVECs and PASMCs in vitro. (**f, g**) Effects of PSS31, NPF, HP, and HPF on the contractile function of PASMCs in hypoxia-injured co-culture system (red: F-actin; blue: DAPI). (**h**) Representative flow cytometry images of intracellular Fluo-4/Ca²⁺ fluorescence intensity in PASMCs. **(i, j)** Western blot analysis of the effect of PSS31, HP, NPF, and HPF on proliferation and contraction-related proteins in hypoxia-injured PASMCs. Data were presented as mean ± SD, *n* = 3. ^*^P < 0.05, ^**^P < 0.01. Statistical significance was calculated *via* one-way ANOVA with Tukey’s test

To investigate the anti-migratory effect of HPF, a transwell co-culture system was utilized to mimic the pulmonary vascular microenvironment (Fig. 3b). Crystal violet staining demonstrated a marked increase in PMVECs migration under hypoxic condition, confirming the promotive effect of hypoxia on endothelial motility. HPF significantly attenuated the migration of hypoxia-injured PMVECs as compared with PSS31, NPF and HP (Fig. 5a, b).

The anti-proliferative capacity of HPF on hypoxia-injured PASMCs was assessed by monitoring Ki67 expression in PASMCs within the same co-culture system. Hypoxia markedly increased the proportion of Ki67-positive PASMCs. PSS31, NPF, HP and HPF attenuated the proportion of Ki67-positive PASMCs, and HPF exhibited the lowest proportion of Ki67-positive PASMCs (Fig. 5c, d).

Pulmonary vasoconstriction represents not only an early hallmark but also a central driver of HPH progression [39]. To investigate modulation effect of HPF on the contraction of PASMCs, a co-culture system of PMVECs and PASMCs was established (Fig. 5e). Phalloidin staining revealed that hypoxia induced pronounced cytoskeletal remodeling in PASMCs, characterized by perinuclear condensation of actin fibers and a shrunken cellular morphology. HPF effectively reversed these structural alterations (5f, g), indicating that HPF had superior efficacy in suppressing PASMC contraction. Since cytosolic free Ca²⁺ is a key regulator of vascular smooth muscle contraction, intracellular Ca²⁺ level was examined using the Fluo-4 AM probe. Flow cytometric analysis demonstrated that hypoxia significantly elevated Fluo-4 fluorescence intensity in PASMCs. Notably, this effect was potentiated by co-culture PASMCs with PMVECs, suggesting that endothelial-derived factors synergistically contributed to Ca²⁺ overload in hypoxia-injured PASMCs. HPF treatment markedly diminished intracellular Ca²⁺ fluorescence, outperforming PSS31, NPF and HP (Fig. 5h, S25). Together, these results demonstrated that HPF relieved vasoconstriction by restoring calcium homeostasis in hypoxia-injured PASMCs.

Western blot analysis further revealed that HPF downregulated cyclin D1, thereby impairing G1-to-S phase progression and inhibiting aberrant proliferation of hypoxia-injured PASMCs. Concurrently, HPF reduced receptor-operated calcium entry by downregulating TRPC6 expression, while upregulating the voltage-gated potassium channel Kv1.5 to promote potassium efflux and membrane hyperpolarization in hypoxia-injured PASMCs (Fig. 5i, j). This hyperpolarized state subsequently closed voltage-dependent calcium channels (VDCCs), further attenuating calcium influx in hypoxia-injured PASMCs.

In summary, these experiments demonstrated that HPF effectively inhibited hypoxia-induced migration of PMVECs as well as hypoxia-induced proliferation and contraction of PASMCs. Through these coordinated actions on multiple pathogenic processes, HPF disrupted core mechanisms driving pulmonary vascular remodeling.

### HPF concentrated in pulmonary vasculature in HPH rats

**Fig. 6:**
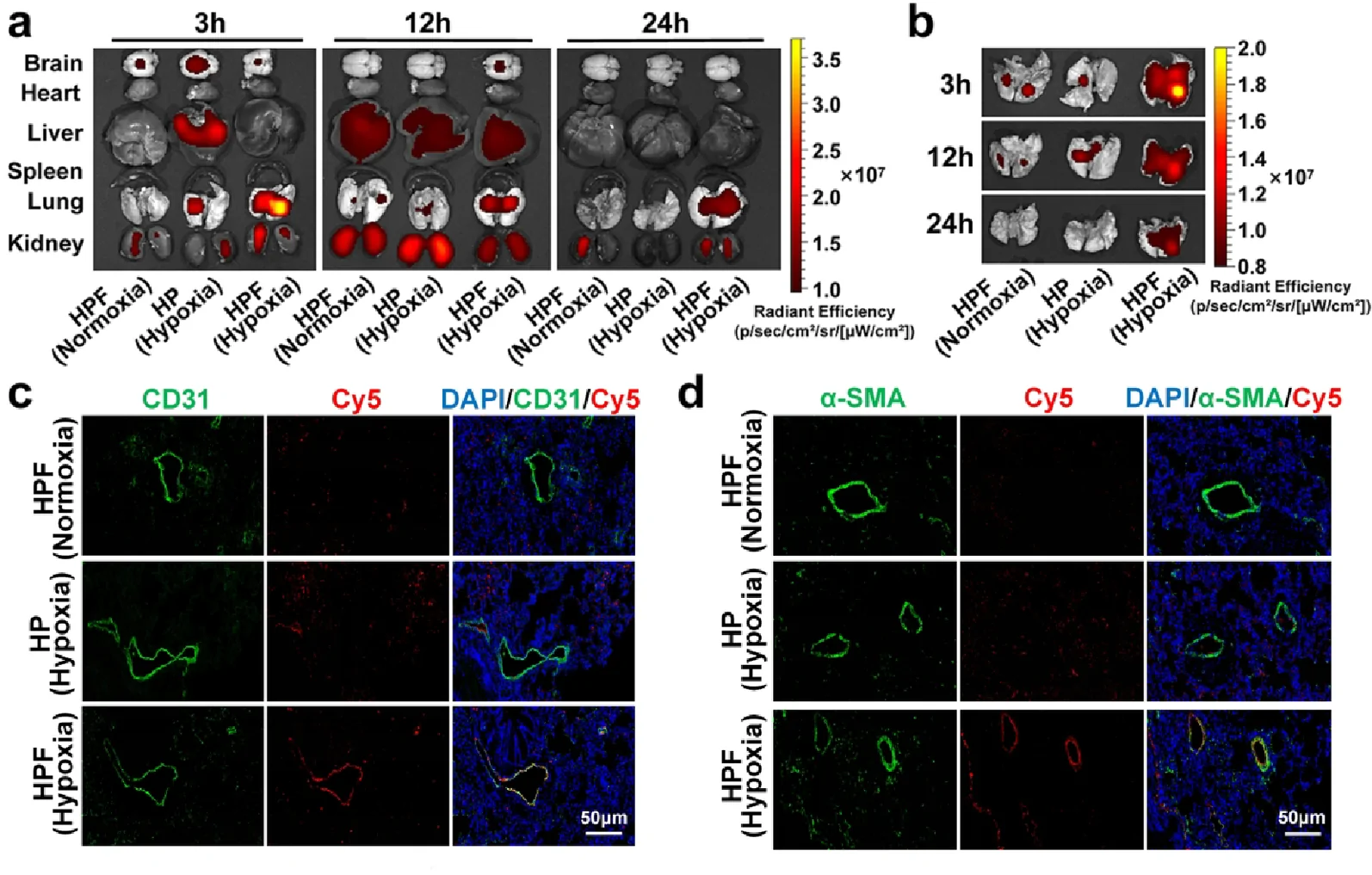
The biodistribution of HPF in HPH rats. (**a**) Fluorescence in intact organs from HPH or normoxic rats at 3, 12 and 24 h after intravenous injection of HPF or HP. (**b**) Fluorescence distribution of HPF and HP in lungs from HPH rats or normoxic rats. (**c**) Immunofluorescence images showing colocalization of Cy5-labelled HP and HPF with pulmonary arterial endothelium in lung tissue sections from normoxic rats or HPH rats (red: Cy5-labelled HPF or HP; green: CD31; blue: DAPI). (**d**) Immunofluorescence images showing colocalization of Cy5-labelled HP and HPF with PASMCs in lung tissue sections from normoxic rats or HPH rats (red: Cy5-labeled HPF or HP; green: α-SMA; blue: DAPI)

The targeting efficiency and retention time of drug delivery system at the lesion site are key determinants for their clinical translation potential [40, 41]. HPF accumulated robustly in the lungs of HPH rats, peaking at 3 h post-injection. The strong fluorescent signal remained detectable in the lungs even at 24 h post-administration. Notably, HPF accumulation in lungs was significantly higher than that of HP. Moreover, HPF accumulation was significantly higher in the lungs in HPH rats than that in normal rats (Fig. 6a, b and Fig.S26, 27). These findings demonstrated that HPF showed more selective accumulation in hypoxia-injured lung tissue than that in normal lung tissue. To further verify the precise vascular targeting of HPF, immunofluorescence was performed on lung sections collected at 3 h post-administration of HPF. In HPH rats treated with HPF, clear colocalization of Cy5 (red) signals with both the endothelial marker CD31 (green) and the smooth muscle marker α-SMA (green) were observed (Fig. 6c, d). This specific co-distribution demonstrated that HPF could actively concentrate in pulmonary endothelial cells and smooth muscle cells in HPH rats.

### HPF inhibited the expression of HIF-1α and the production of ROS in lung tissue in HPH rats

**Fig. 7:**
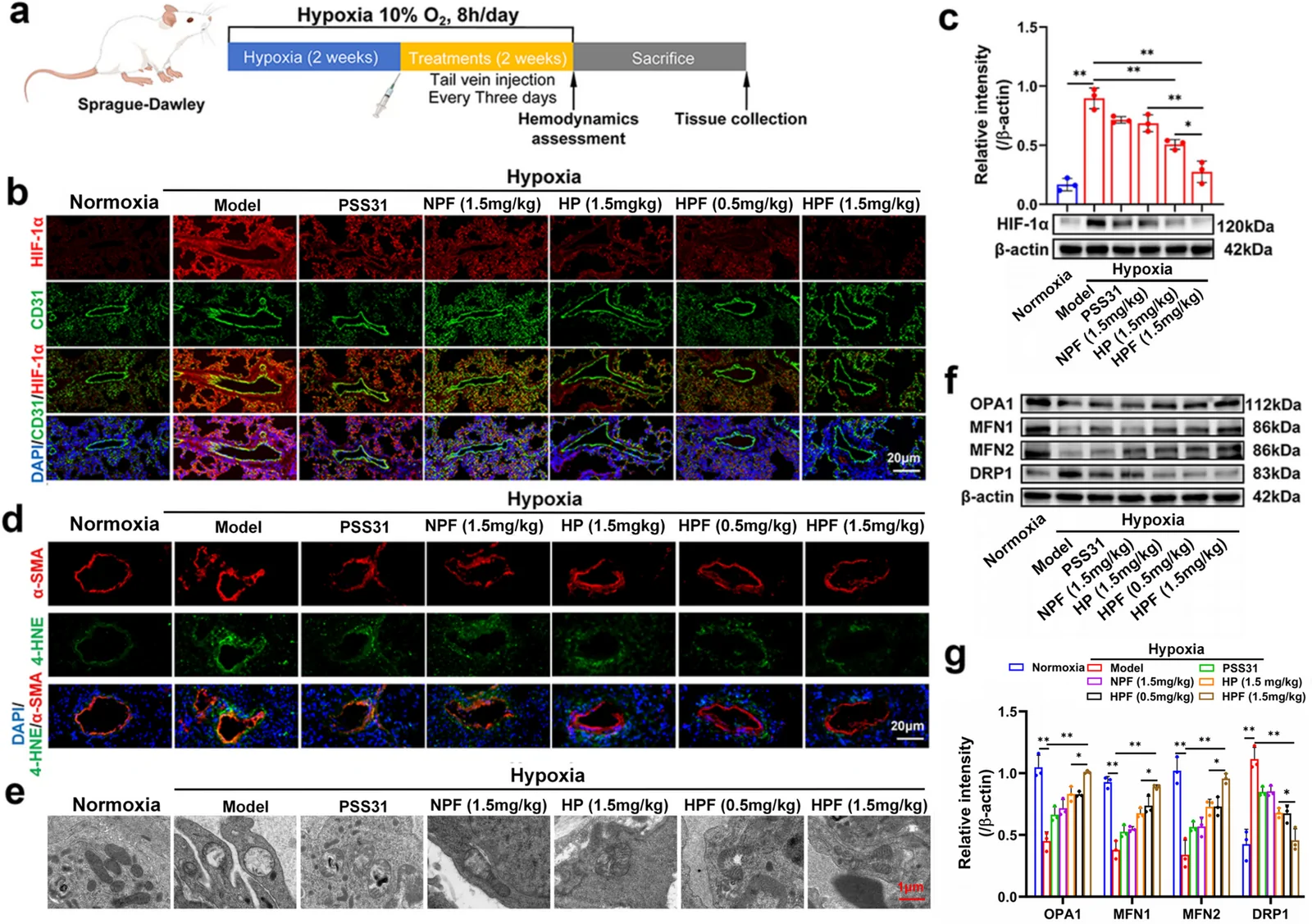
HPF inhibited the expression of HIF-1α and the production of ROS in lung tissue in HPH rats. (**a**) Time schedule of drug administration and therapeutic evaluation on HPH rats. (**b**) Immunofluorescence images showing HIF-1α expression in the pulmonary arterial endothelium of HPH rats or normoxic rats (green: CD31; red: HIF-1α; blue: DAPI). (**c**) HIF-1α expression in rat lung tissues detected via western blot. (**d**) 4-HNE contents in the pulmonary arterial smooth muscle cells of HPH rats or normoxic rats (green: 4-HNE; red: α-SMA; blue: DAPI). (**e**) Transmission electron micrographs of mitochondrial morphology in lung tissue of HPH rats or normoxic rats. NPF indicates siNC/PSS31@Fuco. **(f, g**) Effect of PSS31, NPF, HP, and HPF on the expression of OPA1, MFN1, MFN2 and DRP1 in rat lung tissues. Data were presented as mean ± SD, *n* = 3. ^*^P < 0.05, ^**^P < 0.01. Statistical significance was calculated *via* one-way ANOVA with Tukey’s test

The therapeutic effect of HPF was evaluated in HPH rats following a two-week treatment period during sustained hypoxic exposure. As outlined in Fig. 7a, Sprague-Dawley (SD) rats were randomly assigned to receive intravenous injections of PSS31, NPF, HP, or HPF every three days for two weeks. Immunofluorescence co-staining of HIF-1α and CD31, along with western blot analysis, revealed a marked upregulation of HIF-1α expression in lung tissues in HPH rats, and the expression of HIF-1α was more effectively suppressed by HPF than by HP (Fig. 7b, c).

Dihydroethidium (DHE) staining further demonstrated that HPF significantly reduced ROS level in lungs of HPH (Fig.S28). As a major lipid peroxidation product induced by ROS, 4-Hydroxynonenal (4-HNE) acts as a biomarker for oxidative stress [42, 43]. Immunofluorescent co-staining of 4-HNE and α-SMA confirmed that HPF observably decreased 4-HNE accumulation in pulmonary vascular (Fig. 7d), further verifying the specific alleviative effect of HPF on vascular oxidative injury. TEM unveiled severe ultrastructural damage in pulmonary vascular mitochondria in HPH rats, including swelling, vacuolization, and cristae disruption. In contrast, HPF restored mitochondrial integrity, with clearly defined cristae and double-membrane structures (Fig. 7e). Western blot analysis indicated that HPF dose-dependently downregulated the fission protein Drp1 and upregulated fusion proteins OPA1, MFN1, and MFN2 (Fig. 7f, g), thereby rebalancing mitochondrial dynamics. Collectively, these results systematically demonstrated that HPF downregulated HIF-1α expression while attenuated ROS generation, which subsequently restored mitochondrial structure and functional balance. Through these coordinated actions, HPF disrupted the vicious cycle between HIF-1α activation and ROS generation, indicating its potential role in the treatment of HPH.

### HPF effectively inhibited the progression of HPH in rats

**Fig. 8:**
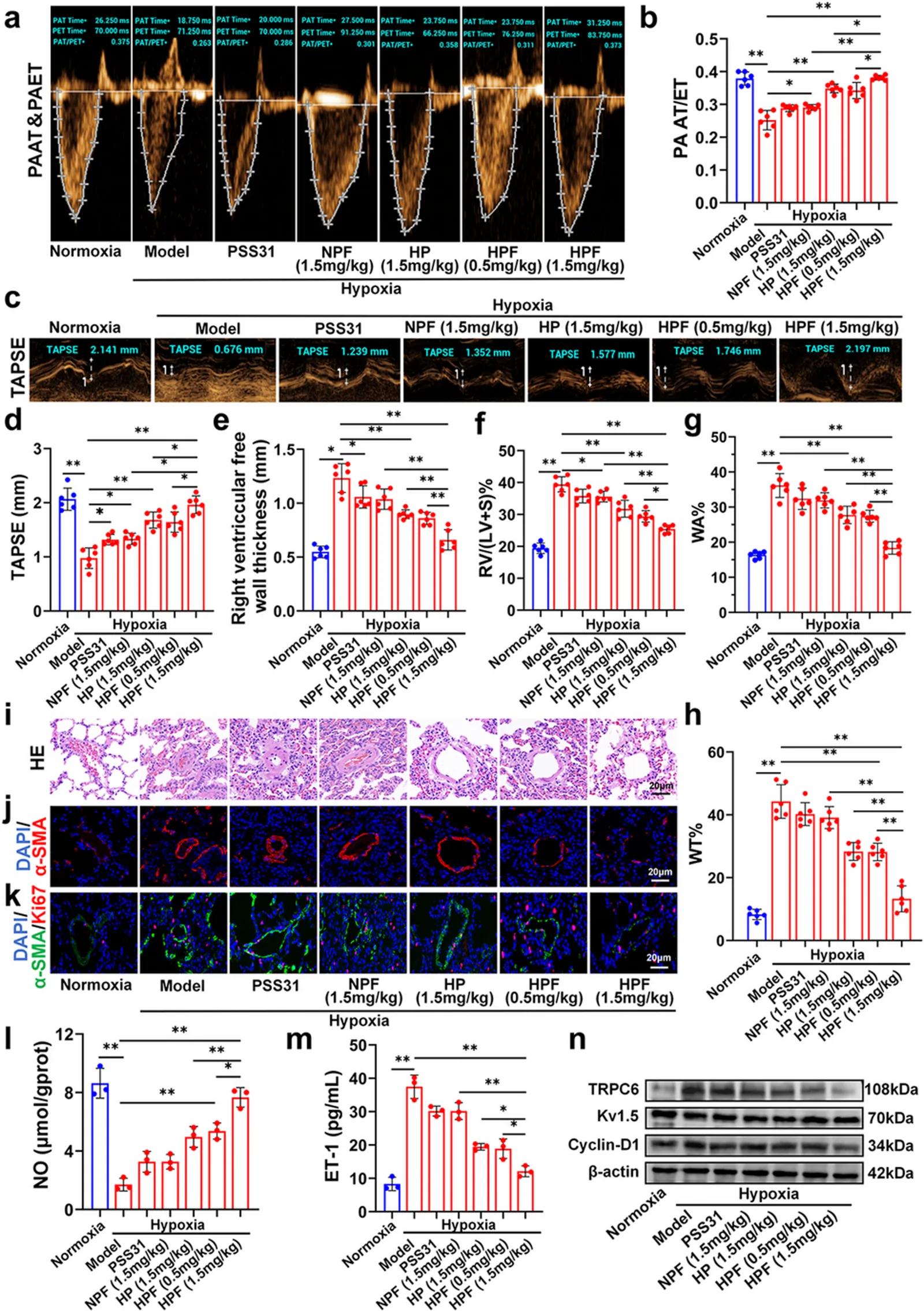
HPF effectively inhibited the progression of HPH in rats. (**a, b)** Echocardiographic measurement of pulmonary artery acceleration time to ejection time ratio (PA AT/ET). Data are mean ± SD. *n* = 6. (**c, d**) Echocardiographic measurement of tricuspid annular plane systolic excursion (TAPSE). Data are mean ± SD. *n* = 6. (**e**) Quantitative analysis of right ventricular free wall thickness (RVWT). (**f**) Quantitative analysis of right ventricular hypertrophy index (RV/(LV + S)%). Data are mean ± SD. *n* = 6. (**g-i**) Histopathological examination was performed to evaluate pulmonary vascular remodeling, the pulmonary artery percent wall area (WA%) and percent wall thickness (WT%) were summarized. Data are mean ± SD. *n* = 6. (**j**) The α-SMA staining of rat lung tissue (red: α-SMA; blue: DAPI). (**k)** The co-staining for Ki67 and α-SMA staining of rat lung tissue (red: α-SMA; green: Ki67; blue: DAPI). (**l, m**) The NO and ET-1 contents in rats lung tissue. Data are mean ± SD. *n* = 3 independent experiments. (**n**) The expression of TRPC6, Kv1.5, and Cyclin-D1 in rats lung tissue. Data were presented as mean ± SD, *n* = 3. ^*^P < 0.05, ^**^P < 0.01. Statistical significance was calculated *via* one-way ANOVA with Tukey’s test

Following the two-week treatment, a comprehensive hemodynamic and functional assessment of HPH rats was conducted. The central pathological feature of HPH is pulmonary vascular remodeling, which narrows the vascular lumen, increases pulmonary vascular resistance, and elevates right ventricular (RV) afterload [44]. In response, the right ventricle undergoes adaptive remodeling, a process manifested as RV hypertrophy that can progress to decompensated failure and further exacerbate pulmonary vascular pathology [45]. Consequently, RV functional parameters serve as critical indicators of both vascular remodeling and overall disease severity. Echocardiographic analysis revealed that HPH rats exhibited a significant reduction in the pulmonary artery acceleration time/ejection time (PA AT/ET) ratio and tricuspid annular plane systolic excursion (TAPSE), alongside a marked increase in RV free wall thickness (RVWT) as compared to normoxia rats. HPF dose-dependently improved PA AT/ET and TAPSE while reducing RVWT, while high-dose HPF demonstrated superior efficacy over the HP (Fig. 8a-e, Fig.S29). Furthermore, the right ventricular hypertrophy index (RV/(LV + S)%) markedly increased in HPH rats, while HPF decreased ventricular in dose-dependent manner (Fig. 8f).

Macrophage serves as the primary cellular source of inflammation-related cytokines [46]. In HPH, massive macrophages infiltrate into injured pulmonary vascular lesions and continuously secrete abundant pro-inflammatory cytokines, thereby triggering and exacerbating local vascular inflammation [47]. Thus, immunofluorescence staining was employed to analyze the polarization status of macrophages surrounding the injured pulmonary arterioles in HPH rats. HPF remarkably facilitated the phenotypic transition to anti-inflammatory M2 macrophages in the periarteriolar regions (Fig.S30). ELISA results further showed that HPF significantly reduced the levels of IL-6, IL-1β and TNF-α in lung tissues, effectively attenuating the inflammatory response in HPH rats (Fig.S31). In addition, as a pivotal transcription factor, HIF-1α can directly mediate the transcriptional expression of pro-inflammatory factors such as TNF-α, IL-6, and IL-1β [48], and the silencing of HIF-1α by HPF effectively inhibited this process. Accordingly, HPF inhibited the secretion of inflammatory factors through a dual mechanism. On the one hand, HPF modulated macrophage polarization to reduce the production and release of pro-inflammatory factors. On the other hand, HPF downregulated HIF-1α expression to block the transcription of inflammation-related genes. Together, these results demonstrated that HPF not only restored hemodynamic function and alleviated maladaptive right heart remodeling but also mitigated pulmonary inflammation, indicating its multimodal therapeutic action in HPH.

The extent of pulmonary vascular remodeling at the histological level was evaluated. The morphological statistical analysis of the distal pulmonary arterioles (100–200 μm in diameter) was conducted using H&E staining, and the wall area percentage (WA%) and wall thickness percentage (WT%) were quantitatively assessed. The results are shown in Fig. 8g-i. Compared with the normoxia rats, the media of the pulmonary arteries became significantly thicker, while the lumen of the vessels was significantly narrowed in HPH rats. Consequently, the WA% and WT% in HPH rats significantly increased. After treatment with HPF, the pulmonary vascular walls became thinner, and the degree of narrowing of the pulmonary arterioles was significantly alleviated in HPH rats. Thus, the WA% and WT% in HPH rats significantly decreased. At the same dosage, the effect of HPF in inhibiting the narrowing of the pulmonary arterioles in HPH rats was significantly stronger than that of HP. Moreover, using α-SMA to label pulmonary vascular smooth muscle cells, and dual-immunofluorescence staining for α-SMA and Ki67 revealed a significant increase in Ki67-positive cells within the pulmonary arterial smooth muscle layer of HPH rats, indicating hyperproliferation of PASMCs in HPH rats. Both HP and HPF treatment could significantly reduce the fluorescence intensity of α-SMA and the Ki67-positive cells in the lung tissues of HPH rats, and HPF had a stronger effect (Fig. 8j-k), indicating HPF effectively reduced the thickness of pulmonary vascular walls and stenosis of the pulmonary arterioles lumen by inhibiting the proliferation of the pulmonary vascular smooth muscle cells.

ET-1 is mainly synthesized by vascular endothelial cells and exerts potent vasoconstrictive effects, its secretion level is positively correlated with the degree of endothelial injury [49, 50]. NO is produced by endothelial nitric oxide synthase (eNOS) and is a key molecule for maintaining vasodilation, inhibiting platelet aggregation, and leukocyte adhesion [51, 52].Therefore, the levels of ET-1 and NO have been widely validated as reliable indicators for assessing endothelial dysfunction in pulmonary vascular diseases [53]. ELISA analysis showed that NO concentration was reduced and ET-1 level was increased in the lung tissues in HPH rats, while HPF dose-dependently increased NO concentration and reduced ET-1 level in the lung tissues in HPH rats (Fig. 8l, m). The above data indicated that the function of PMVECs was improved and the hyperproliferation of PASMCs was inhibited following HPF intervention in HPH rats.

HIF-1α mediated elevation in ROS levels upregulates the expression of TRPC6 and cyclin D1, which subsequently mediates pathological calcium influx and promotes abnormal proliferation of vascular smooth muscle cells. At the same time, HIF-1α mediated elevation in ROS downregulates the expression of Kv1.5, which subsequently disrupts potassium channel homeostasis and induces abnormal vasoconstriction [54–56]. The western blot result indicated that hypoxia significantly upregulated the expression of cyclin D1 and TRPC6 and downregulated the expression of Kv1.5 in rat lung tissues. HPF effectively lowered the expression of TRPC6 and cyclin D1, and enhanced the expression of Kv1.5 in lung tissues of HPH rats (Fig. 8n, Fig. S32). These results demonstrated that HPF regulated the expression of TRPC6, cyclin D1 and Kv1.5 by inhibiting the HIF-1α/ROS signaling pathway, which subsequently ameliorated the dysfunction of PASMCs and alleviated hypoxic pulmonary vascular remodeling.

### Preliminary toxicological evaluation of HPF

The preliminary biosafety of HPF was evaluated in normoxic rats following a two-week treatment period. Results showed no overt pathological changes in the heart, liver, spleen, lungs, or kidneys across all treatment groups (Fig.S33a). The hepatorenal toxicity was further evaluated by measuring plasma biochemical markers. Levels of alanine aminotransferase (ALT), aspartate aminotransferase (AST), blood urea nitrogen (BUN), and creatinine (CREA) remained within normal physiological ranges (Fig.S33b-e), indicating negligible hepatorenal toxicity. Coagulation function was also examined through assays of prothrombin time (PT), activated partial thromboplastin time (APTT), thrombin time (TT), and fibrinogen (FIB). No significant abnormalities were observed in any of these parameters (Fig.S33f-i). In addition, the acute toxicity of HPF was evaluated in normal mice. The results showed that no mice died within 14 days after administration at the maximum soluble concentration of HPF, corresponding to an siRNA dose of 15 mg/kg (with a rat equivalent dose of 7.5 mg/kg). Taken together, these results demonstrated that HPF exhibited preliminary biocompatibility and safety at the administered dosage, supporting its potential for further translational development.

## Conclusions

P-selectin and caveolin-1 were highly expressed in hypoxia-injured PMVECs and PASMCs in HPH rats. Based on this, P-selectin-targeted HPF was developed for siHIF‑1α active delivery to hypoxia-injured PMVECs and PASMCs. Then HPF was taken up by hypoxia-injured PMVECs and PASMCs *via* caveolin-1-dependent endocytosis, effectively avoiding lysosomal degradation of siHIF‑1α and thereby enhancing the silence effect of HIF‑1α. HPF simultaneously downregulated HIF-1α expression and suppressed ROS generation, thereby breaking the vicious cycle between HIF-1α activation and ROS production in HPH rats. This subsequently restored the physiological function of pulmonary microvascular endothelial cells by reducing the migration of endothelial cells, increasing NO level and decreasing ET-1 level. Meanwhile, abnormal proliferation and contraction of pulmonary arterial smooth muscle cells were suppressed by regulating the expression of TRPC6, cyclin D1 and Kv1.5. Ultimately, the pulmonary vascular remodeling, hemodynamic disorders and right ventricular hypertrophy were alleviated by HPF in HPH rats, indicating the potential application of HPF as a multifunctional nanotherapeutic agent for HPH.

Compared to currently available drugs for pulmonary hypertension, our newly developed siHIF‑1α delivery system could more precisely target hypoxia-injured pulmonary vascular cells, rather than acting broadly on systemic vascular cells. This helps effectively avoid drug side effects, such as hypotension and edema. Hypotension and edema are clinically often the biggest barrier to drug accumulation, preventing further control of the pulmonary hypertension condition in patients. Therefore, HPH held greater clinical value. Moreover, the mechanism of existing drugs can only reduce pulmonary vascular resistance by dilating pulmonary blood vessels but can’t fundamentally halt the progression of pulmonary hypertension. In contrast, by targeting the HIF-1α/ROS vicious cycle to inhibit the onset and development of pulmonary hypertension, our approach not only effectively improved pulmonary arterial pressure but also fundamentally suppressed the underlying inflammation associated with pulmonary hypertension, thereby preventing disease progression.

### The limitations and future prospects

At first, right ventricular systolic pressure (RVSP) and mean pulmonary artery pressure (mPAP) sensitively reflect hemodynamic disorders in the early stage of pulmonary hypertension. Therefore, RVSP and mPAP should be concerned in further investigation. Secondly, although this study examined changes in the expression of TRPC6, Kv1.5, and cyclin D1 in whole-lung tissue, these proteins in pulmonary arteries should be received attention in further study. Finally, the long-term in vivo toxicity of HPF should be systematic assessed in further investigation to provide sufficient experimental evidence for its clinical translation.

## Experimental section

### Materials and reagents

O-benzotriazol-1-yl-tetramethyluronium hexafluorophosphate (HBTU), N,N-diisopropylethylamine (DIPEA), Piperazine (PIP), and N, N-dimethylformamide (DMF), were purchased from Chinese Peptide Co., Ltd. (Hangzhou, China). SS31, was purchased from GL Biochem (Shanghai, China). Fucoidan (Fuco), MTT cell growth assay, and 2-deoxy-D-glucose were purchased from Sigma-Aldrich (MA, USA). FAM-siHIF-1α, Cy5-siHIF-1α, and siHIF-1α were purchased from GenePharma Co., Ltd. (Shanghai, China). 3-(propane-2,2-diylbis (thioalkanediyl)) dipropionic acid (TK), and Cy5-Fuco were purchased from Yusi Pharmaceutical Technology Co., Ltd. (Chongqing, China). Chlorpromazine, colchicine, and methyl-β-cyclodextrin were purchased from J&K Scientific Chemical Ltd. (Beijing, China). Lipofectamine 3000, and Fetal bovine serum (FBS) were purchased from Thermo Fisher Scientific Inc. (MA, USA). BCA protein assay kit, and SDS-PAGE sample loading buffer (5×) were purchased from New Cell & Molecular Biotech Co., Ltd. (Suzhou, China). Mitochondrial membrane potential detection kit, ROS assay kit, and mitochondrial superoxide red fluorescent probe were purchased from Med Chem Express Co., Ltd. (Shanghai, China). Lysosomal Tracker red and mitochondrial membrane permeability transition pore detection kit were purchased from Yeasen Biotechnology Co., Ltd. (Shanghai, China). TRITC-Phalloidin fluorescent probes was purchased from Deeyee Biotechnology Co., Ltd. (Shanghai, China). Calcium ion green fluorescent probe, Ribonuclease A (RNase A), RNA loading buffer (4X), and RIPA lysis buffer (strong) were purchased from Beyotime Biotechnology (Shanghai, China). DAPI was purchased from Zhuanzhi Biotechnology Co., Ltd. (Xian, China). Rat NO Colorimetric Assay Kit, and rat ET-1 ELISA kit were purchased from Elabscience Biotechnology Co.,Ltd. (Wuhan, China). Rat ELISA kits for IL-6 and TNF-α were obtained from CUSABIO Co., Ltd. (Wuhan, China). Dulbecco’s modified Eagle’s medium (DMEM), β-actin antibody, 4-HNE antibody, CD86 antibody, CD206 antibody, DAPI, HRP conjugated Goat Anti-Rabbit IgG were purchased from Servicebio Technology Co., Ltd. (Wuhan, China). Caveolin-1 antibody, Drp1 antibody, OPA1 antibody and MFN2 antibody were purchased from Abcam (Cambridge, England). MFN1 antibody, Ki67 antibody, P-selectin antibody, TRPC6 antibody, KCNA5 antibody, Alexa Fluor 488-conjugated anti-rabbit, and Alexa Fluor 594-conjugated anti-mouse were purchased from Proteintech Group (Wuhan, China). HIF-1α antibody, CD31 antibody, α-SMA antibody were purchased from Affinity Biosciences. Co., Ltd. (Suzhou, China).

### Animals

Male Sprague-Dawley (SD) rats (180–220 g) with SPF grade were purchased from the Animal Center of Air Force Medical University. All experiments were approved by Animal Care and Use Committee at Air Force Medical University (IACUC Issue NO-20250156). A rat model of HPH was established by housing the animals in a hypobaric hypoxic chamber simulating an altitude of 5500 m (ambient pressure: 380 mmHg, oxygen concentration: 10%) for 8 h per day. All rats were housed in a standard animal facility maintained at a 12 h dark-light period and allowed free access to food and water.

### Synthesis of PSS31

SS31 was dissolved in a mixed solvent of 1,4-dioxane and water. NaHCO₃ was then added to the reaction system, followed by stirring in an ice bath for 15 min. Afterwards, 9-fluorenylmethyl-N-succinimidyl carbonate was added in batches, and the mixture was stirred at room temperature overnight. An equal volume of deionized water was added to quench the reaction. The product was extracted with ethyl acetate and concentrated under reduced pressure. The target product, compound 1, was obtained after separation and purification by silica gel column chromatography. Accurately weighed quantities of compound 1 (1000 mg, 1.2 mmol) and thioketal (TK) (328 mg, 1.3 mmol) were placed into a round-bottom flask. A sufficient amount of dichloromethane (DCM) was added to dissolve the mixture under stirring at room temperature. Subsequently, O-benzotriazol-1-yl-tetramethyluronium hexafluorophosphate (HBTU) (531 mg, 1.4 mmol) and N, N-diisopropylethylamine (DIPEA) (129 mg, 1.2 mmol) were added. The reaction mixture was stirred overnight at room temperature under a nitrogen atmosphere. After the reaction was confirmed to be complete by thin-layer chromatography (TLC), the mixture was concentrated using rotary evaporation to remove the majority of the solvent. Then an equal volume of deionized water (relative to the DCM used) was added to precipitate product. The precipitate was collected by filtration, yielding the crude product. The crude product was purified by recrystallization using petroleum ether (PE)/DCM to obtain compound 2.

Accurately weighed quantities of compound 2 (1096 mg, 1.0 mmol) and compound 3 (948 mg, 1.1 mmol) were placed into a round-bottom flask. A sufficient amount of dichloromethane (DCM) was added to dissolve the mixture under stirring at room temperature. Subsequently, HBTU (455 mg, 1.2 mmol) and DIPEA (129 mg, 1.0 mmol) were added. The reaction mixture was stirred overnight at room temperature under a nitrogen atmosphere. The compound 4 was purified by using the method described in compound 2.

Accurately weighed compound 4 (2328 mg, 1.2 mmol) was dissolved in a sufficient amount of 20% piperazine (PIP) in N, N-dimethylformamide (DMF). The mixture was stirred at room temperature for 2 h. After the reaction was confirmed to be complete by TLC, the reaction mixture was concentrated by rotary evaporation to remove the majority of the solvent. Then, 20 mL DCM was added, and the mixture was stirred thoroughly. The mixture was then concentrated under reduced pressure. This washing procedure (adding 20 mL DCM, stirring, and concentrating) was repeated three times. The resulting product after concentration was purified by recrystallization with PE/DCM to obtain compound 5.

Accurately weighed quantities of compound 5 (1496 mg, 1.0 mmol) and compound 2 (1206 mg, 1.1 mmol) were placed into a round-bottom flask. A sufficient amount of DCM was added to dissolve the mixture under stirring at room temperature. Subsequently, HBTU (455 mg, 1.2 mmol) and DIPEA (129 mg, 1.0 mmol) were added. The reaction mixture was stirred overnight at room temperature under a nitrogen atmosphere. The compound 6 was purified by using the method described in compound 2.

Accurately weighed compound 6 (4383 mg, 1.2 mmol) was dissolved in a sufficient amount of 20% PIP in DMF. The mixture was stirred at room temperature for 2 h. After the reaction was confirmed to be complete by TLC, the reaction mixture was concentrated by rotary evaporation to remove the majority of the solvent. Then, 20 mL DCM was added, and the mixture was stirred thoroughly. The mixture was then concentrated under reduced pressure. This washing procedure (adding 20 mL DCM, stirring, and concentrating) was repeated three times. The resulting product after concentration was purified by recrystallization with PE/DCM to obtain compound 7. The precise molecular weights of the target compound 7 were determined by TOF MS. The structures of compounds 1–7 are shown in Fig.S2.

### Cell culture and treatment

Primary PMVECs and PASMCs were isolated from 4-week-old male SD rats. Briefly, lung periphery tissues and pulmonary artery medial layers were minced into 1 mm³ fragments and explanted in culture dishes. Migrated cells were maintained in DMEM supplemented with 20% FBS and 1% penicillin-streptomycin at 37 °C under 5% CO₂ and 21% O₂. Second-passage cells were characterized by immunofluorescence staining for the endothelial marker CD31 (PMVECs) and the smooth muscle marker α-SMA (PASMCs). For experiments, cells were exposed to either normoxic environment (21% O₂) or hypoxic environment (5% O₂) [57–59].

### Western blot

Protein samples were separated by SDS-PAGE and transferred to PVDF membranes. After blocking with 5% skim milk, the membranes were probed with specific primary antibodies overnight at 4 °C, followed by incubation with HRP-conjugated secondary antibodies. Blots were developed using an ECL detection reagent, and band intensities were quantified after normalization to a loading control (β-actin).

### siHIF-1α sequence screening

Three different siRNA sequences (Tab.S1) targeting rat HIF-1α were transfected into PMVECs using Lipofectamine 3000 to identify the most effective candidate. Second-passage PMVECs were seeded in 6-well plates at a density of 2 × 10⁵ cells per mL and cultured until ~70% confluent. Cells were then subjected to transfection under hypoxic conditions (5% O₂) for 48 h. HIF-1α expression levels were detected by western blot.

### Preparation and characterization of HPF

HP complex was formed by vortexing PSS31 and siHIF-1α−2310 in DEPC water for 30 s, followed by a 30 min incubation. The optimal ratio between PSS31 and siRNA was determined by agarose gel electrophoresis. HPF were then prepared by adding the HP complex dropwise to a Fuco solution (in DEPC water).

The particle size and potential of HP and HPF were measured by laser particle size analyzer (Malvern, Zetasizer Nano ZSE), and the stability of HP and HPF in PBS or in DMEM containing 10% FBS within 6 days were also evaluated. The adsorption of FAM-siHIF-1α (green) and Cy5-Fuco (red) was detected by flow cytometer (Beckman, BF25288, U.S.A), and the morphology of HPF was observed by transmission electron microscope (TEM, HT7700, Hitachi) and scanning electron microscope (SEM, ThermoFisher Quattro S, Thermo Fisher Scientific). The release of SS31 from HPF over time in H₂O₂ solutions at different concentrations was detected using a liquid chromatography-triple quadrupole mass spectrometer (Agilent 1290 + AB SCIEX 3200 QTRAP, USA). Agarose gel electrophoretic analysis was used to examine the stability of HPF against RNase enzyme and its ROS-responsive siHIF-1α release characteristics. Finally, the hemolysis effect of HPF was detected by using 2% rat erythrocyte suspension.

### Immunofluorescence assay

Cell samples grown on glass coverslips were fixed with 4% paraformaldehyde for 15 min at room temperature. Tissue samples were fixed in 4% paraformaldehyde, embedded in paraffin, and sectioned at 5 μm thickness. Following fixation, all samples were permeabilized with a solution containing 0.3% Triton X-100 and blocked by 5% normal serum from the host species of the secondary antibody for 1 h. Samples were then incubated overnight at 4 °C with primary antibodies diluted in blocking solution. After washing, the samples were incubated with appropriate fluorophore-conjugated secondary antibodies for 1 h at room temperature. Nuclei were counterstained with DAPI. Finally, the slides were mounted with an anti-fade mounting medium. Images were acquired using laser scanning confocal microscopy (LSCM, FV10, Olympus), and fluorescence intensity was quantified with ImageJ software.

### Flow cytometry

Cells were harvested and washed twice with cold PBS. For cell surface staining, cells were resuspended in PBS and incubated with fluorochrome-conjugated primary antibodies for 30 min on ice in the dark. Following staining, cells were washed twice with PBS and resuspended in PBS. Appropriate isotype controls and unstained cells were included in each experiment. Data acquisition was performed on a flow cytometer, and a minimum of 10,000 events per sample were collected. Subsequent analysis was conducted using FlowJo software.

### MTT assay

Cells were seeded in 96-well plates at a density of 2 × 10^4^ cells per well and allowed to adhere overnight. Following the desired experimental treatments, the culture medium was replaced with fresh medium containing 5 mg/mL MTT reagent, and the cells were incubated for 4 h at 37 °C. Subsequently, the medium was carefully removed, and the formed formazan crystals were dissolved in 200 µL of dimethyl sulfoxide (DMSO). The absorbance of the solution was measured at 490 nm using a microplate reader. Cell viability was calculated relative to untreated control cells.

### Detection of subcellular distribution of HP and HPF

Second-passage PMVECs and PASMCs were collected and uniformly seeded into 24-well plates at a volume of 500 µL per well with a cell density of 5 × 10^4^ cells/mL, followed by 24 h of routine culture. Afterwards, the original medium was replaced with serum-free medium, and the cells were cultured under hypoxic condition for another 24 h. Subsequently, the cells were incubated with FAM-labeled HP and HPF solutions (the final concentration of FAM-siHIF-1α was 75 nmol/L) under hypoxia for 1 h and 4 h respectively. Lysosomes were specifically labeled by Lysotracker (Green) staining for 30 min in accordance with the manufacturer’s instructions. Cells were fixed with 4% paraformaldehyde, and nuclei were counterstained with DAPI. The subcellular distribution of HP and HPF around lysosomes was observed *via* LCSM. Pearson’s correlation coefficient was further calculated to quantify the lysosomal colocalization degree.

### In vitro trans-endothelial barrier model

Primary rat PMVECs (passage 2) were seeded into the upper chambers of 24-well transwell plates at a density of 2 × 10⁴ cells per well in 200 µL of complete medium. The formation of a confluent endothelial barrier was confirmed by measuring the trans-endothelial electrical resistance (TEER) using a Millicell ERS volt-ohm meter. The model was considered established when TEER values stabilized at approximately 250 Ω·cm². PASMCs were seeded in the lower chambers at the same density (2 × 10⁴ cells per well in 600 µL) and cultured for 24 h. Subsequently, the medium in both chambers was replaced with serum-free DMEM, and the co-culture system was maintained under normoxic environment (21% O₂) or hypoxic environment (5% O₂) conditions for 24 h.

### Serum protein adsorption assay

HPF or HP was incubated in DMEM containing 10% FBS for 24 h. Then, the nanoparticles were isolated from serum via centrifugation (13,680 × g, 15 min, 4 °C). After being extensively washed with deionized water, the nanoparticles were mixed with SDS-PAGE loading buffer. A 10% FBS sample was run in parallel as a control. After electrophoresis, gels were stained with Coomassie Blue. Finally, gels were de-stained prior to imaging.

### Intracellular ROS assay

Second-passage adherent PMVECs or PASMCs were seeded into 24-well plates at a density of 5 × 10⁴ cells per mL (500 µL per well) and cultured for 24 h. Subsequently, the culture medium was replaced with serum-free medium, and the cells were subjected to hypoxic conditions for another 24 h. Following this, the medium was changed to serum-free DMEM containing PSS31, HP, NPF, or HPF (with siHIF-1α at a concentration of 75 nmol/L), and the cells were maintained under hypoxia for an additional 24 h. After treatment, the cells were washed with PBS and incubated with 10 µM DCFH-DA in PBS (500 µL per well) for 20 min at 37 °C in the dark. Following three washes with PBS, the cells were fixed with 4% paraformaldehyde and the nuclei were counterstained with DAPI. DCF fluorescence was visualized and quantified using LSCM.

### Mitochondrial superoxide measurement

Following PBS washes, cells were incubated with 10 µM MitoSOX^™^ Red reagent (300 µL) for 25 min at 37 °C. After thorough washing, cells were fixed with 4% paraformaldehyde, stained with DAPI, and imaged by LSCM to detect red fluorescence intensity.

### mPTP assay

Cells were washed and incubated with 5 µM Calcein-AM (30 µL) for 25 min at 37 °C. Then, 10 µL CoCl₂ solution was added and incubation continued for an additional 20 min. After PBS washes and fixation, calcein green fluorescence was observed by LSCM.

### Mitochondrial membrane potential assay

Cells were stained with 20 µg/mL JC-1 (300 µL) for 20 min at 37 °C. After fixation and DAPI staining, the mitochondrial membrane potential was evaluated by measuring the red/green fluorescence intensity ratio using LSCM.

### Cell migration assay

Second-passage PMVECs and PASMCs were seeded into the upper chambers of 24-well transwell plates (0.8 μm pore size) at a density of 2 × 10⁴ cells per well. After attachment, the medium was replaced with serum-free DMEM containing PSS31, HP, NPF, or HPF (the concentration siHIF-1α was 75 nmol/L). Cells were then cultured under normoxic or hypoxic conditions for 24 h. Following incubation, cells were fixed with 4% paraformaldehyde and stained with 0.1% crystal violet for 30 min. After thorough washing, migrated cells were visualized under a microscope. For quantification, stained cells were dissolved in 30% acetic acid, and the absorbance of the eluate was measured at 570 nm. The relative migration rate was calculated based on the OD570 value.

### Ki-67 proliferation assay

Cells were fixed with 4% paraformaldehyde for 15 min at room temperature and permeabilized with 0.3% Triton X-100 for 10 min. After blocking with 5% normal goat serum for 1 h, samples were incubated overnight at 4 °C with anti-Ki-67 primary antibody diluted in blocking solution. Following PBS washes, cells were incubated with appropriate fluorophore-conjugated secondary antibody for 1 h at room temperature. Nuclei were counterstained with DAPI for 5 min. Coverslips were mounted using anti-fade mounting medium. Ki-67-positive cells were visualized by LSCM and quantified by counting positively stained nuclei in at least five random fields per sample, with results expressed as the percentage of Ki-67-positive cells.

### In vivo treatment study

Male SD rats of similar body weight were acclimatized for one week under standard housing conditions. The rats were then randomly assigned to normoxic or hypoxic groups and maintained for 2 weeks to establish the hypoxic pulmonary hypertension model. Drug administration commenced via tail vein injection on day 2 post-hypoxia exposure and was repeated every 3 days for 2 weeks. Throughout the treatment period, rats were exposed to hypoxic conditions for 8 h daily. Treatment efficacy was evaluated upon completion of the regimen.

### Biodistribution of HPF

Following the treatment period, Cy5-labeled HP or HPF were prepared using Cy5-conjugated siHIF-1α. The nanoparticles were administered to HPH rats via tail vein injection. Rats were euthanized at 3, 12, and 24 h post-injection, and major organs (lungs, heart, liver, brain, spleen, and kidneys) were collected. The distribution of nanoparticles in each organ was visualized and analyzed using an in vivo imaging system (Vieworks, Smart-LF).

### Hemodynamics and right ventricular hypertrophy assessment

Following treatment completion, rats were anesthetized with isoflurane, depilated, and positioned supine on a thermostatic heating plate (37 °C). Hemodynamic parameters were evaluated using a high-resolution ultrasound imaging system (Z60, Mindray). Heart rate was maintained at 335 ± 15 beats/min with continuous ECG monitoring. Pulmonary artery acceleration time/ejection time (PAAT/ET), tricuspid annular plane systolic excursion (TAPSE), and right ventricular free wall thickness (RVWT) were respectively measured using B-mode, M-mode, and pulsed-wave Doppler imaging. After echocardiography, rats were euthanized, and heart were collected. The heart was dissected to isolate the right ventricle (RV), left ventricle (LV), and interventricular septum (S). Following removal of atria and great vessels, each compartment was weighed separately. The degree of right ventricular hypertrophy was expressed as the RV/(LV + S) ratio (Fulton’s index).

### Pulmonary vascular remodeling assessment

Left lung tissues from each group were fixed in 4% paraformaldehyde for 24 h. Then, it was dehydrated and embedded in paraffin. After that tissue sample was sectioned into 5 μm thickness. Sections were stained with hematoxylin and eosin (H&E) and examined under a light microscope. Pulmonary vascular remodeling was assessed using image analysis software (Image-Pro Plus v6.0, Media Cybernetics). Six peripheral distal pulmonary arteries per sample with external diameters of 100–200 μm were randomly selected. The internal and external diameters, wall thickness, and vascular area were measured. The percentage of medial wall thickness (WT%) and wall area (WA%) were calculated as following equation.

$$\begin{aligned}&\rm WT\,\%=(2 \times medial\,wall\,thickness\,/\,external\,diameter)\\& \quad\times100\%\end{aligned}$$

$$\rm WA\,\% = (medial\,wall\,area\,/\,total\,vascular\,area)\,\times\,100\%$$

### Detection of ROS in lung tissue

Fresh lung tissues were cryo-sectioned and the sections were stained with DAPI for nuclear counterstaining. After three washes with TBST, the sections were incubated with dihydroethidium (DHE) staining solution (1:400 dilution) for 1 h at 37 °C. Following TBST washes, sections were mounted and immediately imaged using LSCM. ROS levels were quantified by measuring DHE fluorescence intensity.

### Assessment of mitochondrial morphology in lung tissue

Following treatment, lung tissues were rapidly collected and fixed in pre-cooled (4 °C) electron microscopy fixation buffer for 2 h. Small tissue blocks (2 mm ×2 mm) were prepared, followed by embedding and ultrathin sectioning. Mitochondrial morphology in the lung tissues was examined using a transmission electron microscope (HT7800, Hitachi, Japan).

### Endothelin-1 quantification

Lung tissue homogenates samples were prepared for analysis. The concentration of endothelin-1 (ET-1) was determined using a commercial ELISA kit according to the manufacturer’s protocol. Briefly, standards and samples were added into antibody-coated wells and incubated for 2 h at room temperature. After washing, a biotinylated detection antibody was added, followed by incubation with streptavidin-HRP conjugate. Tetramethylbenzidine (TMB) substrate was used for color development, and the reaction was stopped with sulfuric acid. Absorbance was measured at 450 nm, and ET-1 concentrations were calculated according to standard curve.

### Nitric oxide measurement

NO levels in lung tissues were determined using a commercial nitric oxide assay kit based on the Griess reaction. Tissue homogenates were centrifuged at 12,000 × g for 15 min at 4 °C, and the supernatants were collected for analysis. Following the manufacturer’s protocol, 50 µL of each sample was mixed with 100 µL of Griess reagent I and 100 µL of Griess reagent II in a 96-well plate. The mixture was incubated for 10 min at room temperature in dark room. Absorbance was measured at 540 nm using a microplate reader. Nitric oxide concentrations were calculated according to standard curve and normalized to total protein content determined by BCA assay.

### Inflammatory cytokines quantification

Lung tissues were homogenized in cold PBS (1:9 w/v) containing protease inhibitors. The homogenates were centrifuged at 12,000 × g for 20 min at 4 °C, and the supernatants were collected for analysis. Concentrations of IL-6 and TNF-α were measured using commercial ELISA kits according to the manufacturers’ protocols. Briefly, 100 µL of each standard and sample were added to antibody-coated wells and incubated for 2 h at room temperature. After washing, biotinylated detection antibodies were added, followed by incubation with streptavidin-HRP conjugate. TMB substrate was used for color development, and the reaction was stopped with stop solution. Absorbance was measured at 450 nm within 30 min. Cytokine concentrations were determined according to standard curve.

### In vivo safety assessment

Rats were randomly divided into six groups and administered normal saline, PSS31, NPF, HP, low-dose HPF, or high-dose HPF via tail vein injection every 3 days for 2 weeks. For serum biochemistry, blood samples were collected and centrifuged at 4,000 × g for 15 min at 4 °C. Serum levels of ALT, AST, BUN, and CREA were measured using an automated biochemical analyzer (Chemray 800, China). For coagulation analysis, EDTA-anticoagulated blood was centrifuged at 1,500 × g for 15 min at 4 °C, and plasma PT, APTT, TT, and FIB were determined with an automated coagulation analyzer (RAC1830, China). Major organs (brain, heart, liver, spleen, lungs, and kidneys) were harvested, fixed, and processed into paraffin sections. H&E staining was performed, and whole-slide imaging was conducted using a Pannoramic MIDI II scanner (3DHISTECH) for histopathological evaluation. Acute toxicity was evaluated in normal mice following the method described in reference [60], and the toxic effects of HPF nanoparticles were assessed by monitoring the mortality of the mice.

### Statistical analysis

Data are presented as the mean ± standard deviation (SD). All statistical analyses and graphing were performed using GraphPad Prism software. For the statistical comparison between the two groups, the two-tailed Student’s t test was performed. For pairwise multiple comparisons, the one-way analysis of variance (ANOVA) with Tukey’s post-hoc test was used. For comparisons between other groups and a specific control group, the one-way ANOVA with Dunnett’s test was applied. A *P* value of less than 0.05 was considered statistically significant.

## Supplementary Information

Supplementary Material 1.

Supplementary Material 2.

## Data Availability

All data supporting the findings of this study are available within the paper and its Supplementary Information.
