# Supplementary material for: Hypoxia-injured pulmonary vascular targeting and microenvironment-responsive nanoparticle to treat hypoxic pulmonary hypertension
Source: J Nanobiotechnology. 2026 Jul 13;24:936. doi: 10.1186/s12951-026-04680-z (PMC13644269; doi:10.1186/s12951-026-04680-z)

Original Western bot images

Group： [Rat lung tissues]
Normoxia Hypoxia

Fig 1b and Fig S10.

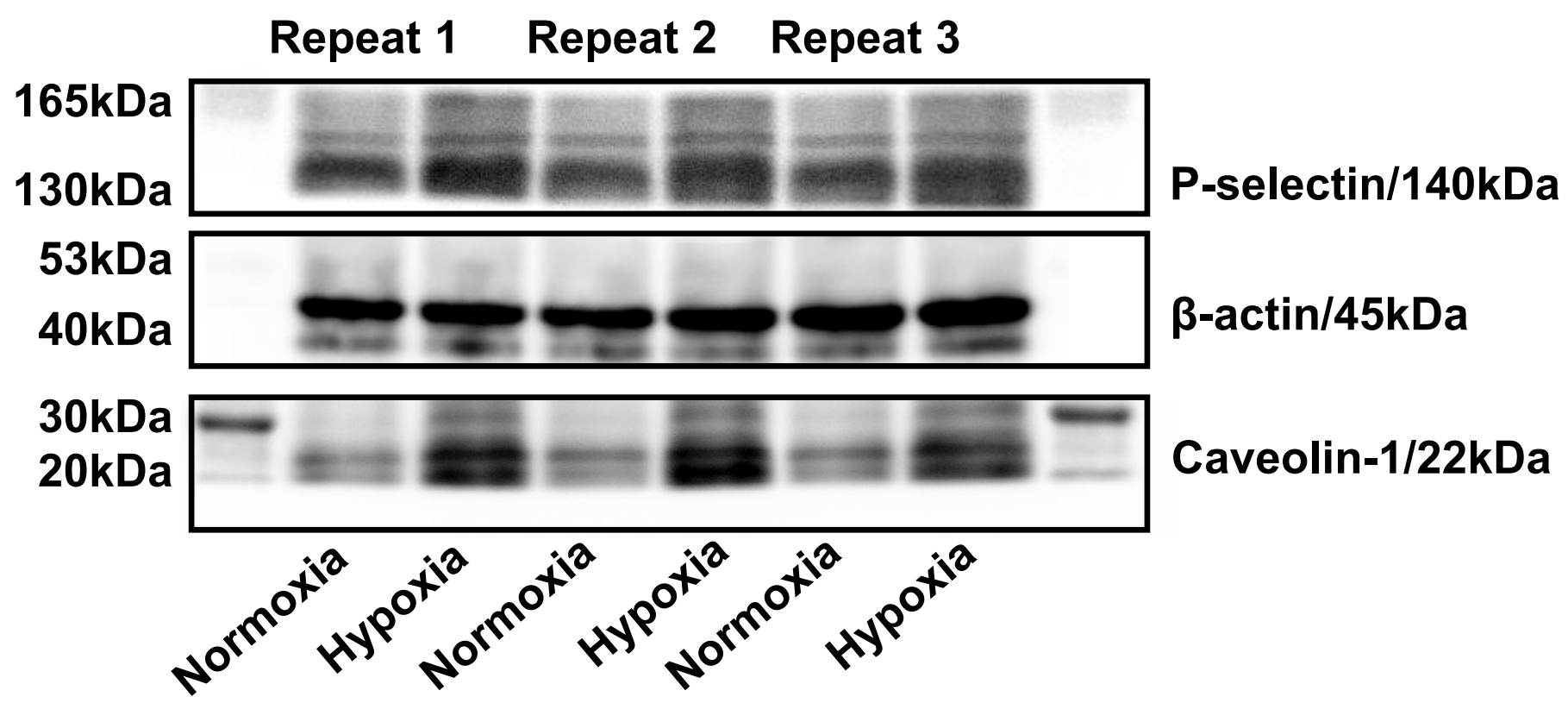

Group： [PMVECs and PASMCs]
Normoxia(24h、48h)
Hypoxia(24h、48h)

Fig 2b.

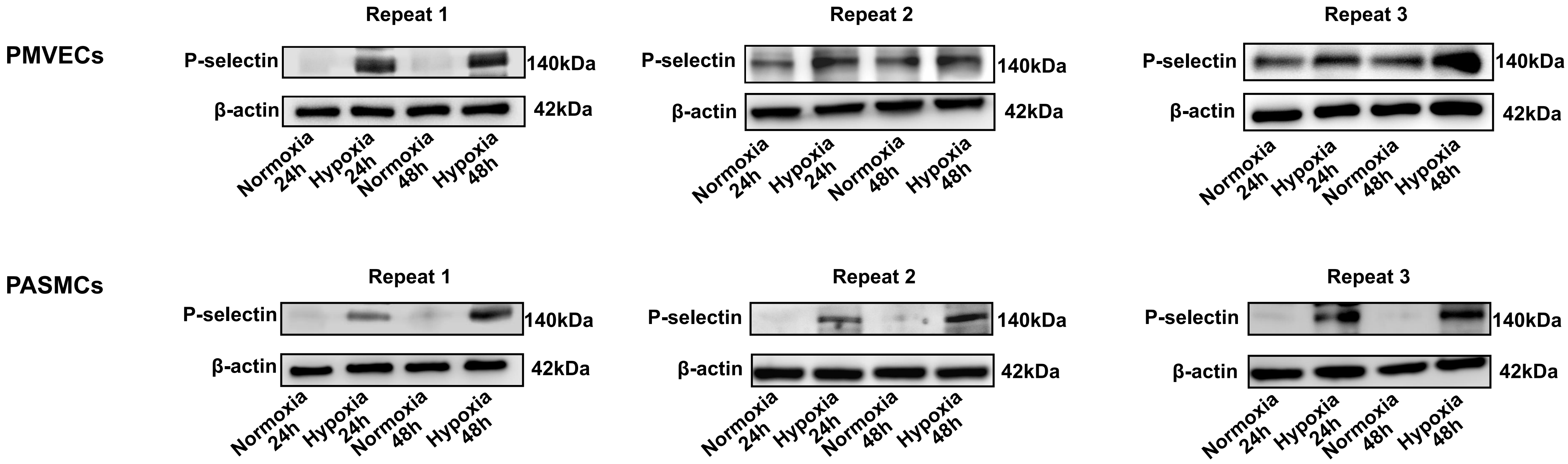

Group： [PMVECs and PASMCs]
Normoxia Hypoxia

Fig 2c.

PMVECs

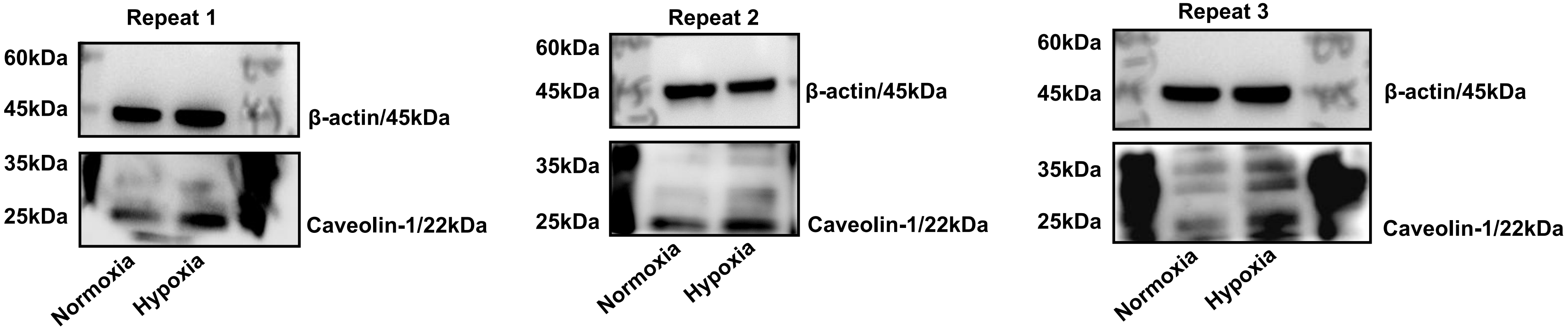

PASMCs

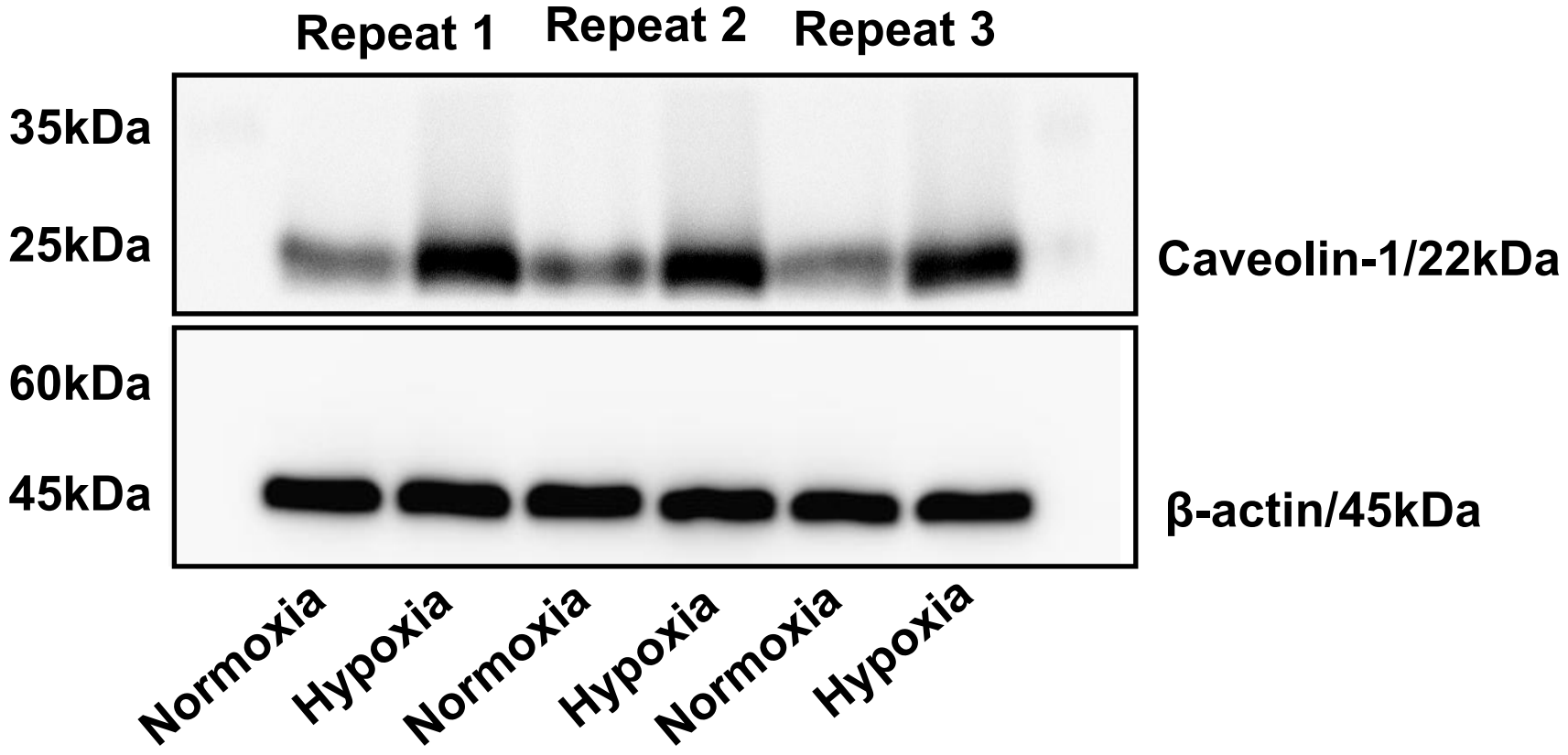

Group： [PMVECs and PASMCs]
Normoxia Hypoxia（24h、48h）

Fig 4a and Fig 4b.

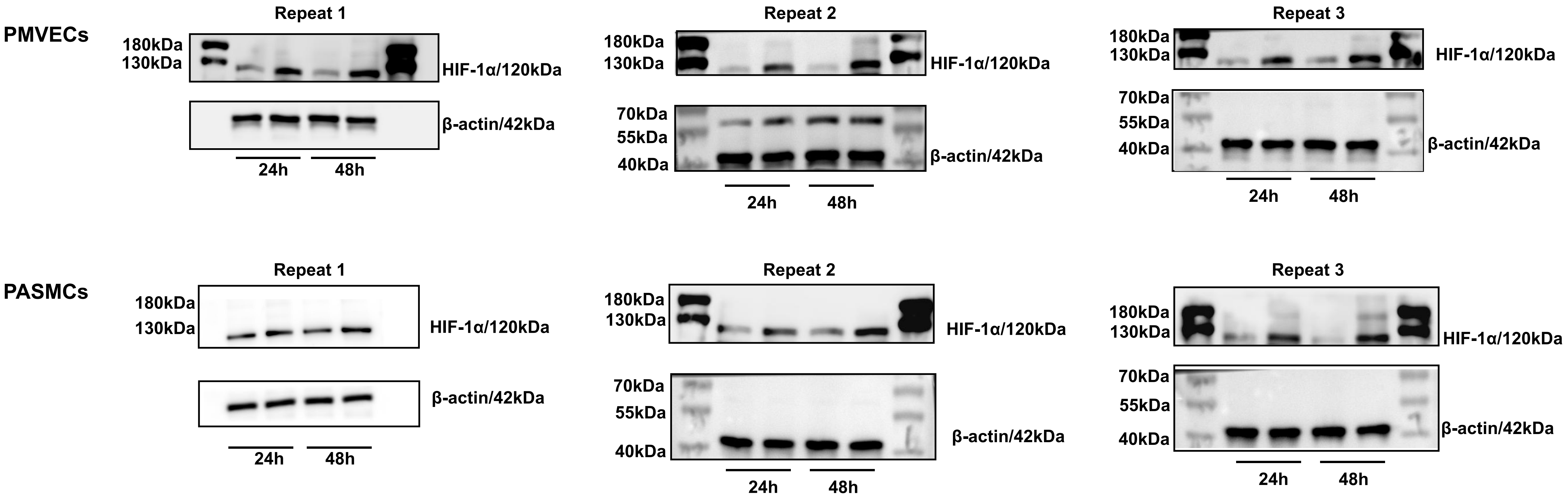

Group： [PMVECs and PASMCs]

Model、NPF、H@lipo3000、HP、HPF

Fig 4c.

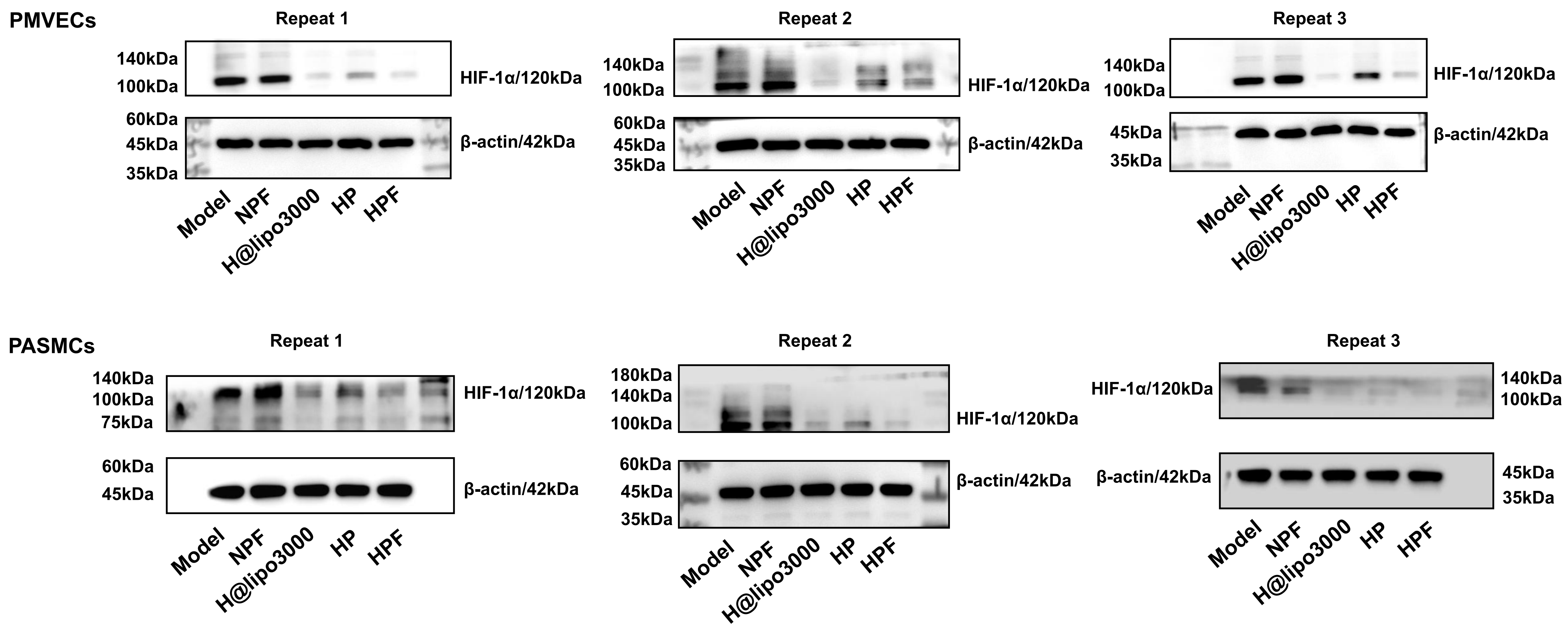

Group：[PMVECs and PASMCs]

Normoxia、Model、PSS31、NPF、HP、HPF

Fig 4i and Fig 4j

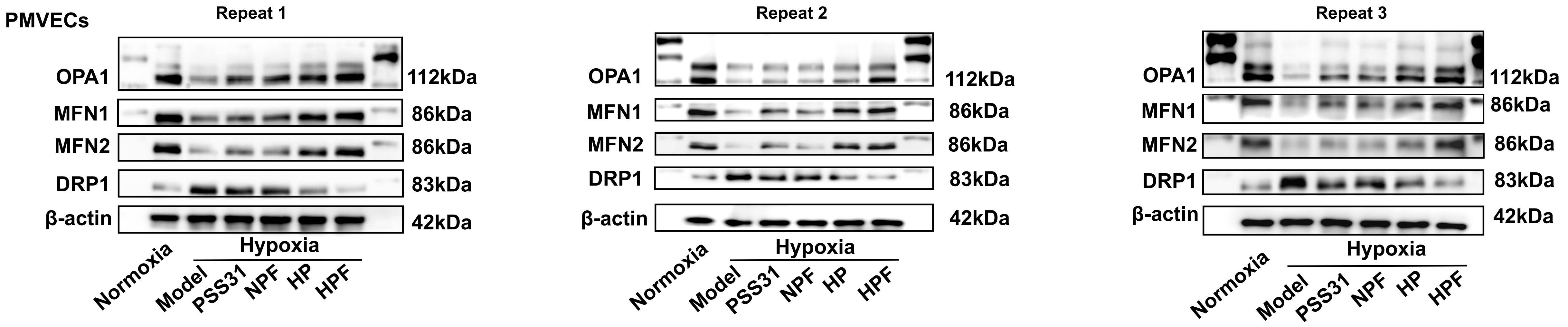

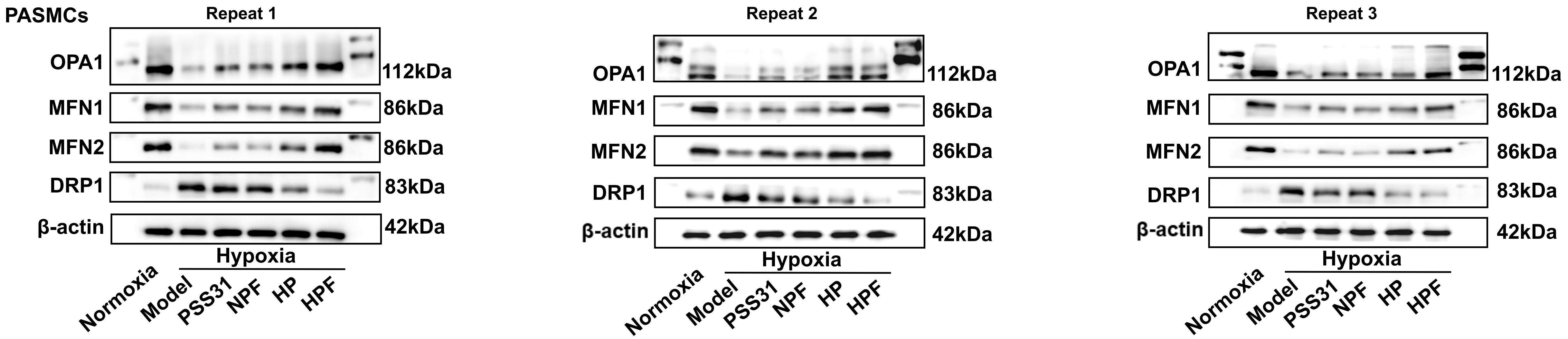

Group： [PASMCs]

Normoxia、Model、PSS31、NPF、HP、HPF

Fig 5i.

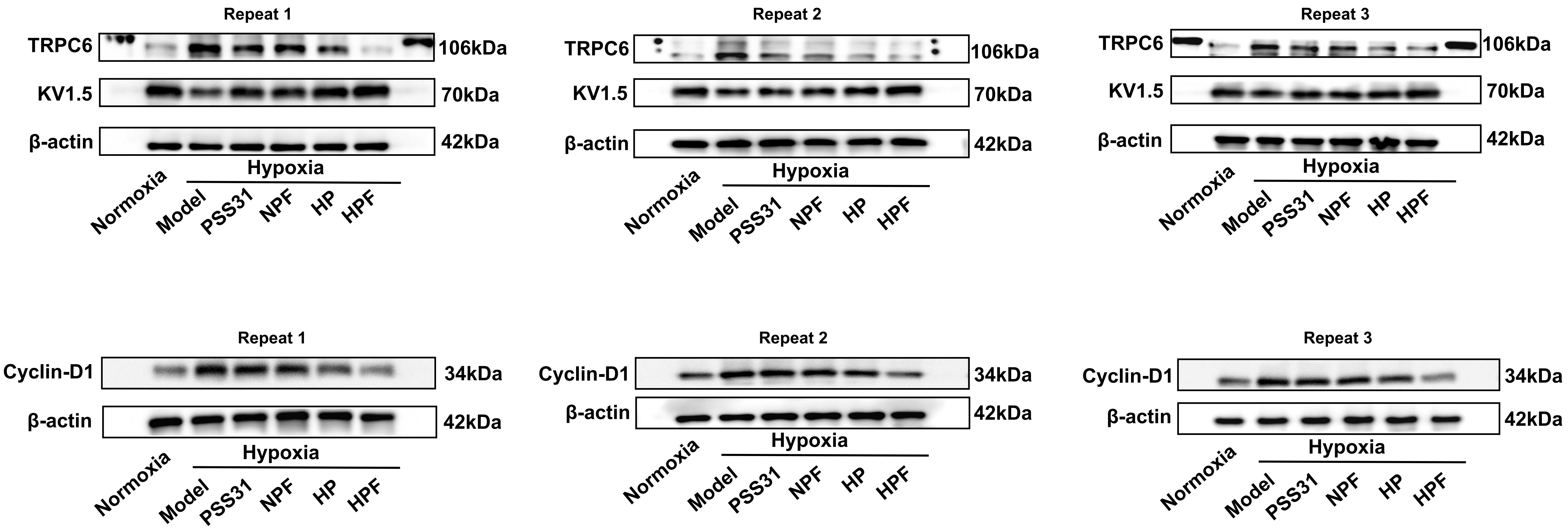

Group：[Rat lung tissues]
Normoxia、Model、PSS31、
NPF(1.5mg/kg)、HP (1.5mg/kg)、
HPF (1.5mg/kg)

Fig 7c.

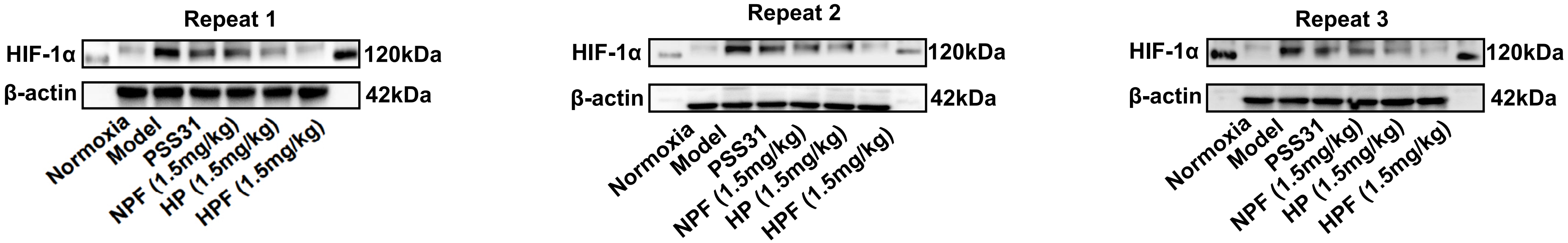

Fig 7f.

Group: [Rat lung tissues]

Normoxia、Model、PSS31、NPF(1.5mg/kg)、

HP (1.5mg/kg)、HPF (0.5mg/kg)、HPF、 (1.5mg/kg)

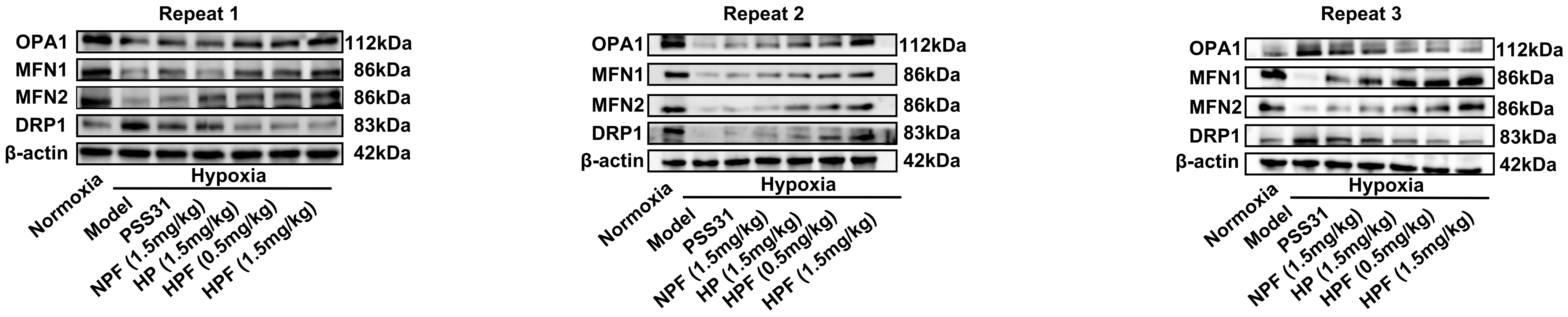

Group： [Rat lung tissues]
Normoxia、Model、PSS31、NPF(1.5mg/kg)、
HP (1.5mg/kg)、HPF (0.5mg/kg)、HPF (1.5mg/kg)

Fig 8n.

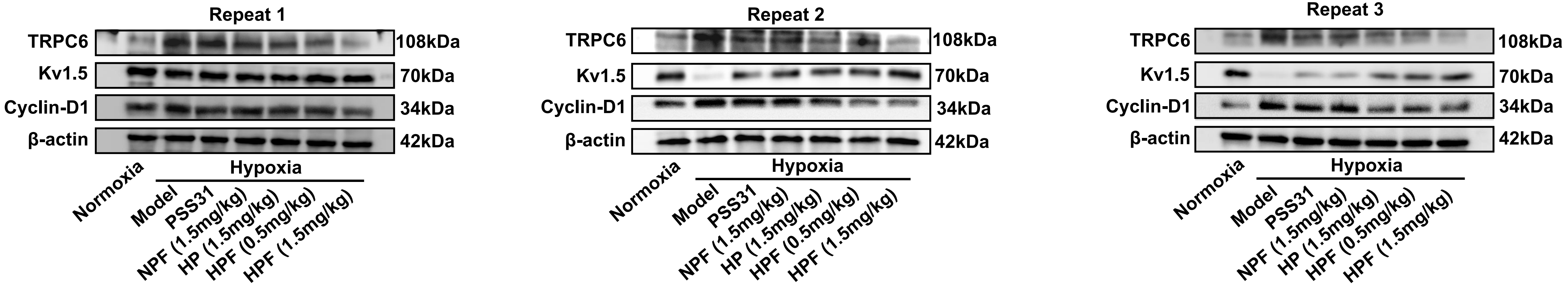

Group： [PMVECs]
Normoxia、Model、si-HIF-1α-1925、
si-HIF-1α-2910、si-HIF-1α-2833、si-HIF-1α-2310

Fig S1.

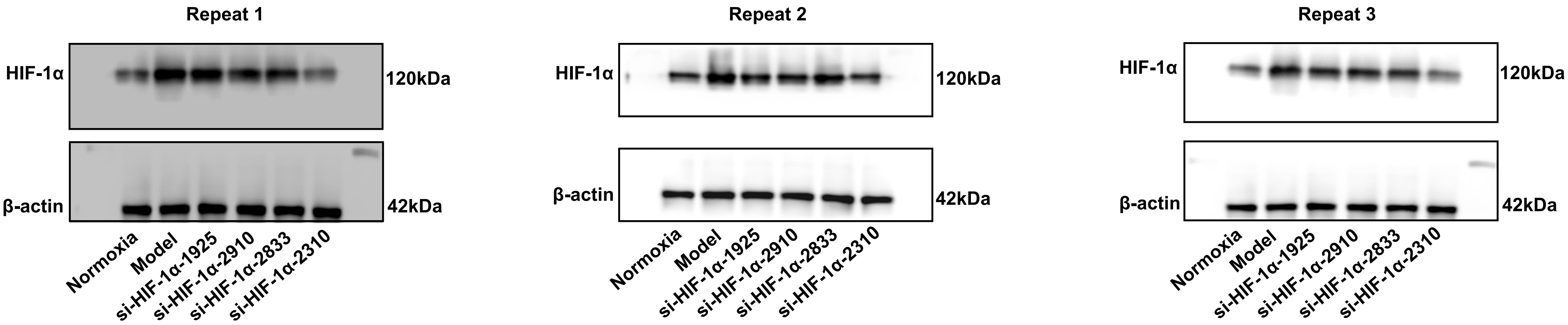

Original gel images of agarose gel electrophoresis

Fig 1a.

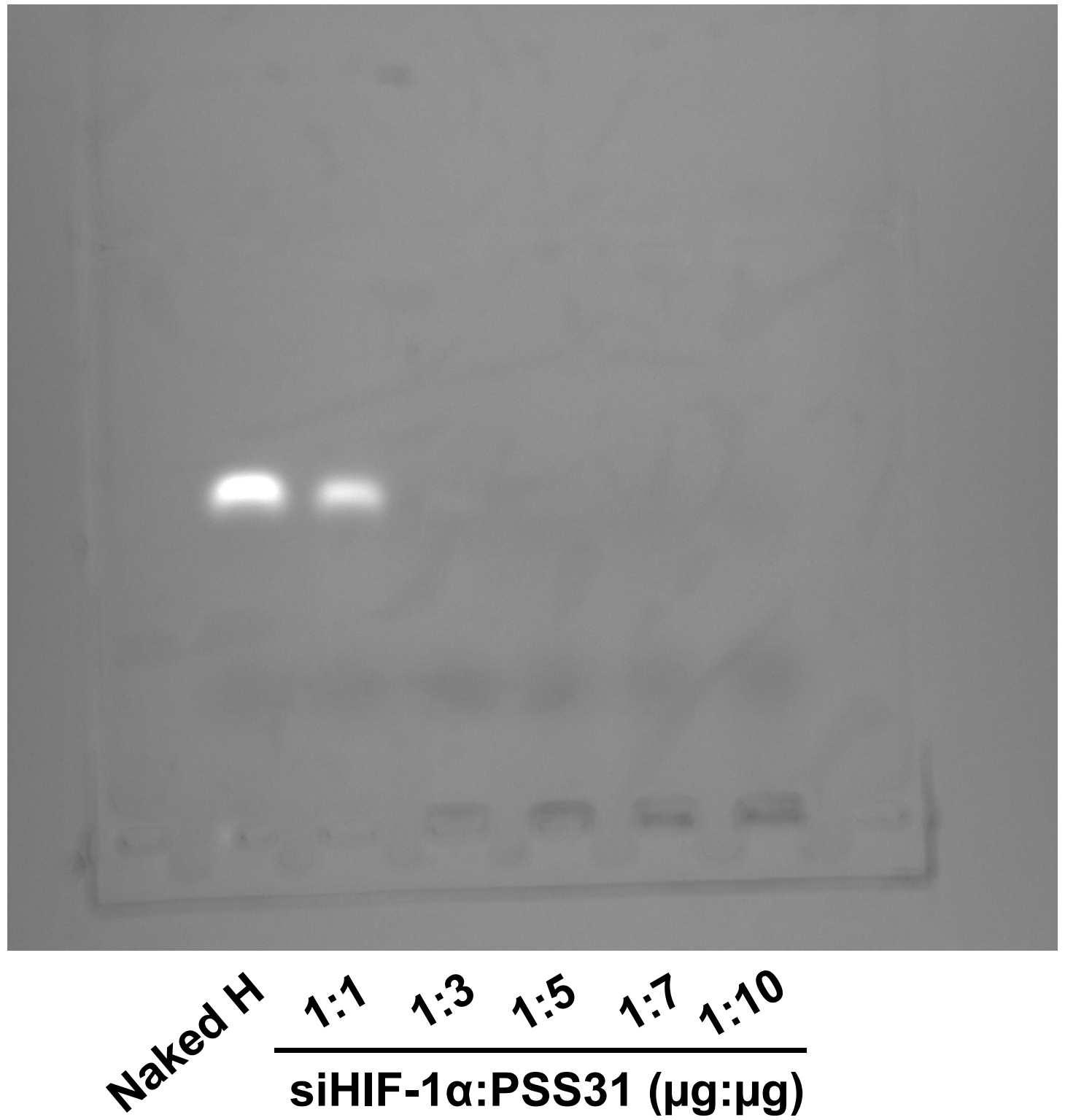

Fig 1i.

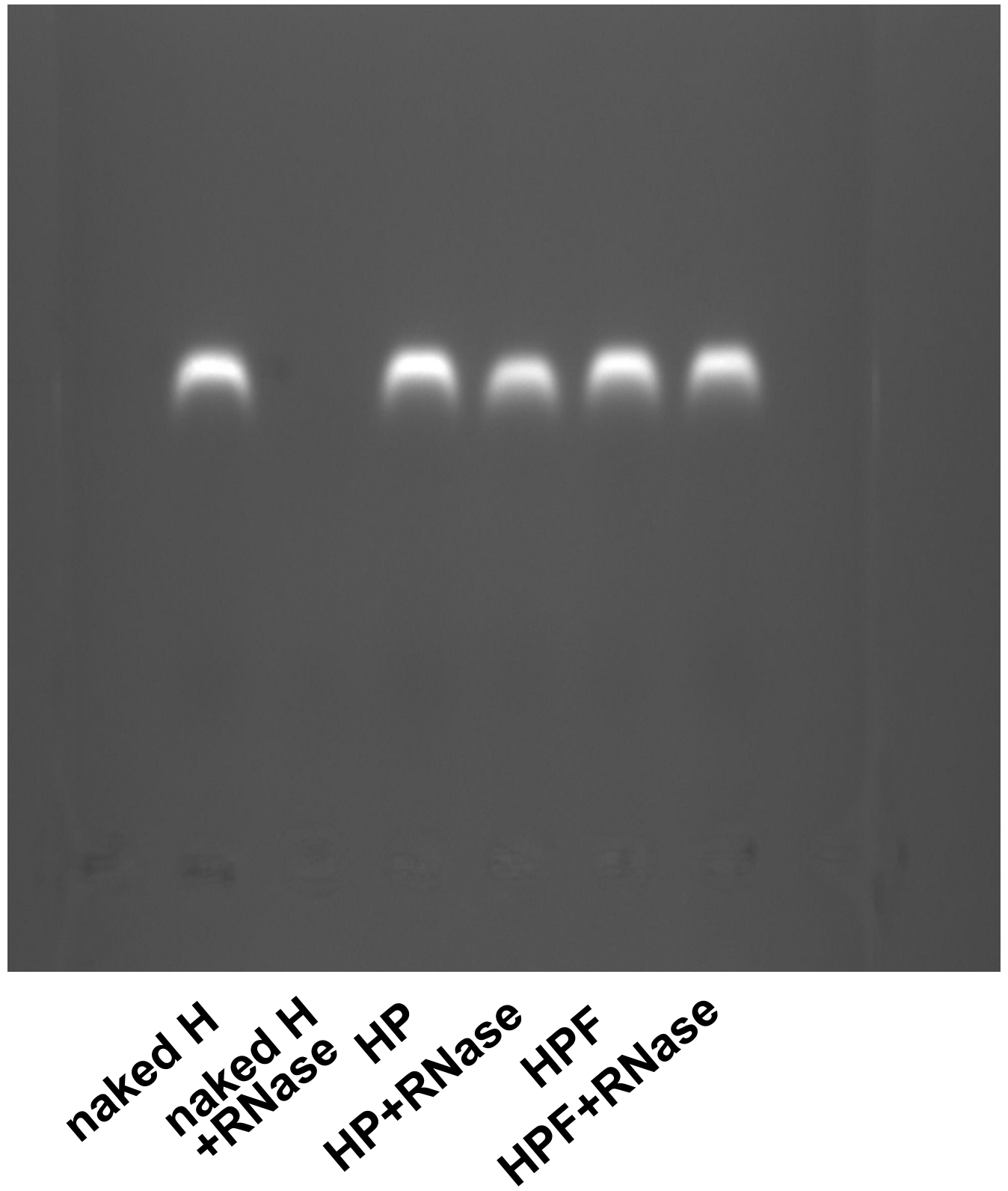

Fig 1k.

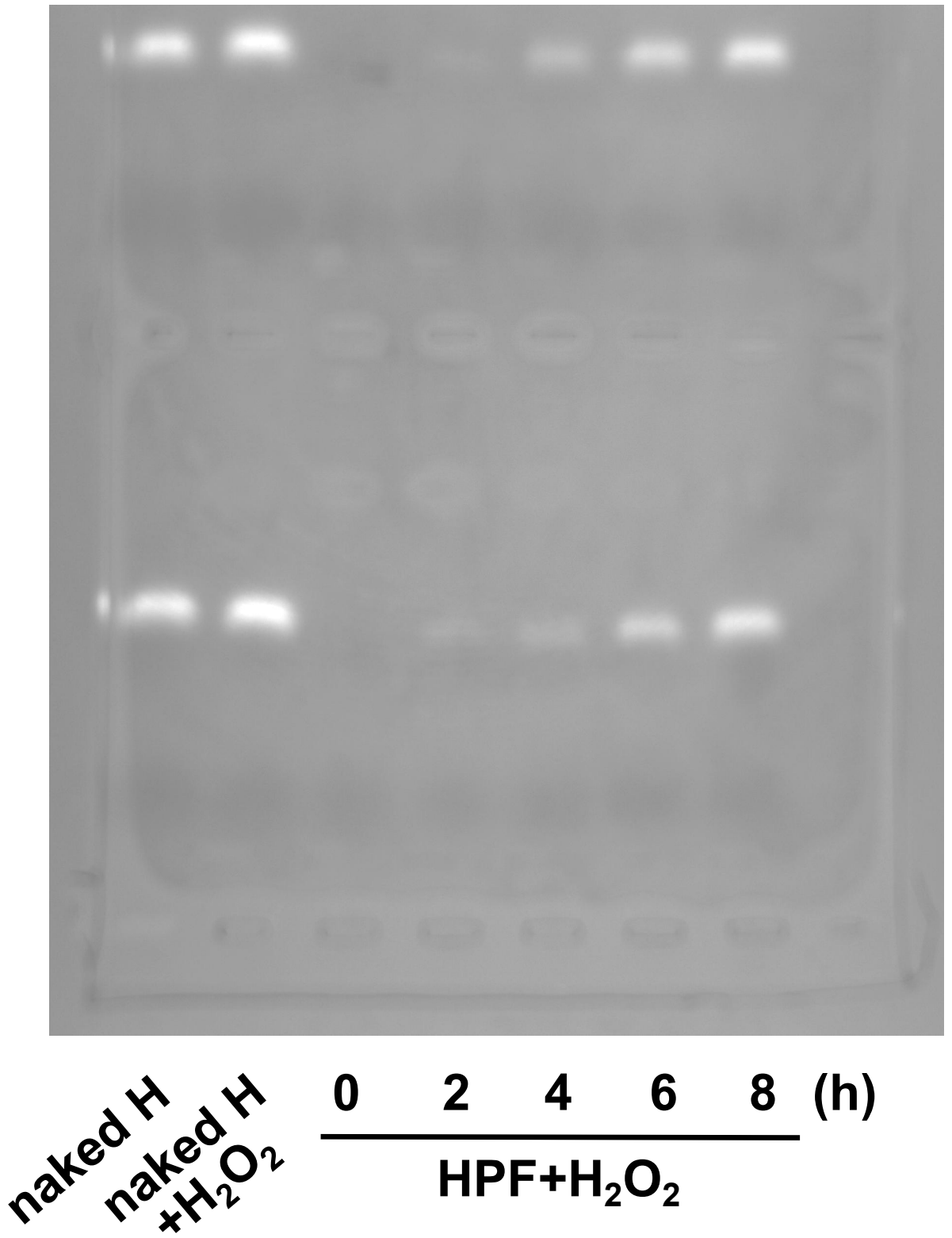

Supplement: Supplementary file 1 [file 12951_2026_4680_MOESM2_ESM.pdf]
