# Supplementary material for: Hypoxia-injured pulmonary vascular targeting and microenvironment-responsive nanoparticle to treat hypoxic pulmonary hypertension
Source: J Nanobiotechnology. 2026 Jul 13;24:936. doi: 10.1186/s12951-026-04680-z (PMC13644269; doi:10.1186/s12951-026-04680-z)
Supplement: Supplementary file 2 [file 12951_2026_4680_MOESM1_ESM.docx]

**Supplementary information**

Tab.1 Detailed information of siHIF-1α sequences designed against rat from different sources.

| **Gene name** | **Sequence** | |
| --- | --- | --- |
|  | sense（5'-3'） | antisense（5'-3'） |
| HIF-1α-2910 | GAGCUUUGGAUCAAGUUAATT | UUAACUUGAUCCAAAGCUCTT |
| HIF-1α-2833 | GCUGACCAGUUACGAUUGUTT | ACAAUCGUAACUGGUCAGCTT |
| HIF-1α-2310 | CCACCACUGAUGAAUCAAATT | UUUGAUUCAUCAGUGGUGGTT |
| HIF-1α-1925 | GAUGGAAGCACUAGACAAATT | UUUGUCUAGUGCUUCCAUCTT |

**Fig. S1** The HIF-1α silencing efficiency of different siHIF-1α sequences. Data were presented as mean±SD, n=3. ^*^*P*<0.05, ^**^*P*<0.01. Statistical significance was calculated *via* one-way ANOVA with Tukey’s test.

**Fig. S2** Synthesis strategy of PSS31.

**Fig. S3** TOF-MS spectrum of PSS31.

**Fig. S4** The particle size of HP (a), zeta potential of HP (b) and HPF (c).

**Fig. S5** The particle size (a) and PDI (b) of the HP and HPF in DMEM containing 10% FBS. Data were presented as mean±SD, n=3.

**Fig. S6** Hemolytic effects of HPF on rat erythrocytes (a) and quantitative analysis (b). Data were presented as mean±SD, n=3.

**Fig. S7** The effect of HPF on the viability of PMVECs (a) and PASMCs (b). Data were presented as mean±SD, n=5.

**Fig. S8** Identification of primary PMVECs and PASMCs by immunofluorescence staining. (a) The expression of CD31 in primary PMVECs. (b) The expression of α-SMA in primary PASMCs (green: CD31; red: α-SMA; blue: DAPI).

**Fig. S9** Cellular uptake of HP and HPF by PMVECs and PASMCs. (a) The uptake of HP by PMVECs in normoxic or hypoxic environment detected by flow cytometry. (b) The uptake of HPF by PASMCs in normoxic or hypoxic environment detected by flow cytometry. (c) The uptake of HP by PASMCs in normoxic or hypoxic environment detected by flow cytometry. Data were presented as mean±SD, n=3. ^*^*P*<0.05, ^**^*P*<0.01. Statistical significance was calculated *via* one-way ANOVA with Tukey’s test.

**Fig.S10** Expression of caveolin-1 in lung tissues of HPH rats. Data were presented as mean±SD, n=3. *^**^P*<0.01. Statistical significance was calculated *via* unpaired two-tailed Student’s t test.

**Fig. S11** Quantitative analysis of P-selectin (a) and caveolin-1 (b) expression in PMVECs and PASMCs detected by western blot. Data were presented as mean±SD, n=3. ^**^*P*<0.01. Statistical significance was calculated *via* one-way ANOVA with Tukey’s test (a) or unpaired two-tailed Student’s t-test (b).

**Fig. S12** The uptake of HP and HPF by PMVECs in the present of various inhibitors. (a) Cellular uptake counts of HP by PMVECs in normoxic environment. (b) Cellular uptake counts of HP by PMVECs in hypoxic environment. (c) Cellular uptake counts of HPF by PMVECs in normoxic environment. (d) Cellular uptake counts of HPF by PMVECs in hypoxic environment. Data were presented as mean±SD, n=3. ^**^*P*<0.01. Statistical significance was calculated *via* one-way ANOVA with Dunnett's test.

**Fig. S13** The uptake of HP or HPF by PASMCs in the present of various inhibitors. (a) Cellular uptake counts of HP by PASMCs in normoxic environment. (b) Cellular uptake counts of HP by PASMCs in hypoxic environment. (c) Cellular uptake counts of HPF by PASMCs in normoxic environment. (d) Cellular uptake counts of HPF by PASMCs in hypoxic environment. Data were presented as mean ± SD, n=3. ^**^*P*<0.01. Statistical significance was calculated *via* one-way ANOVA with Dunnett's test.

**Fig. S14** Effect of HPF on TEER values. Data were presented as mean±SD, n=3. Statistical significance was calculated *via* unpaired two-tailed Student’s t-test. ns: no significant difference.

**Fig. S15** Accumulation of FAM-HPF in PASMCs after penetration endothelial barrier detected by flow cytometry. Data were presented as mean±SD, n=3. ^**^*P*<0.01. Statistical significance was calculated *via* one-way ANOVA with Tukey’s test.

**Fig. S16** LSCM images showing accumulation of FAM-HPF in PASMCs after penetration endothelial barrier (green: FAM-labelled HPF; blue: DAPI).

**Fig. S17** Uptake of HP and HPF by co-cultured RAW264.7, PMVECs, and PASMCs. (a) Schematic illustration of the co-culture model of RAW264.7, PMVECs, and PASMCs *in vitro*. (b) Quantitative analysis of HP uptake by RAW264.7, PMVECs and PASMCs. (c) Quantitative analysis of HPF uptake by RAW264.7, PMVECs, and PASMCs. Data were presented as mean±SD, n=3. *^*^P*<0.05, ^**^*P*<0.01. Statistical significance was calculated *via* one-way ANOVA with Tukey’s test. ns: no significant difference.

**Fig. S18** The particle size (a) and PDI (b) of HPF in DMEM solution containing 10% FBS.

**Fig. S19** Immunofluorescence staining images showing the expression of HIF-1α in PMVECs after treatment with HPF under hypoxic environment (green: HIF-1α; blue: DAPI).

**Fig. S20** Semi-quantitative analysis of relative DCF-green fluorescence intensity in hypoxia-injured PMVECs (a) and PASMCs (b) treated with PSS31, HP, NPF and HPF. Data were presented as mean±SD, n=3. ^*^*P*<0.05, ^**^*P*<0.01. Statistical significance was calculated *via* one-way ANOVA with Tukey’s test.

**Fig. S21** Semi-quantitative analysis of relative MitoSOX Red fluorescence intensity in hypoxia-injured PMVECs (a) and PASMCs (b) treated with PSS31, HP, NPF and HPF. Data were presented as mean±SD, n=3. ^*^*P*<0.05, ^**^*P*<0.01. Statistical significance was calculated *via* one-way ANOVA with Tukey’s test.

**Fig. S22** Semi-quantitative analysis of relative Calcein fluorescence intensity in hypoxia-injured PMVECs (a) and PASMCs (b) treated with PSS31, HP, NPF, and HPF. Data were presented as mean±SD, n=3. ^*^*P*<0.05, ^**^*P*<0.01. Statistical significance was calculated *via* one-way ANOVA with Tukey’s test.

**Fig. S23** The ratio between red and green fluorescence intensity showing the effects of PSS31, HP, NPF and HPF on mitochondrial membrane potential in hypoxia-injured PMVECs (a) and PASMCs (b). Data were presented as mean±SD, n=3. ^*^*P*<0.05, ^**^*P*<0.01. Statistical significance was calculated *via* one-way ANOVA with Tukey’s test.

**Fig. S24** Statistical analysis of the expression of mitochondrial dynamics-related protein in hypoxia-injured PMVECs (a) and PASMCs (b) treated with PSS31, NPF, HP and HPF. Data were presented as mean±SD, n=3. ^*^*P*<0.05, ^**^*P*<0.01. Statistical significance was calculated *via* one-way ANOVA with Tukey’s test.

**Fig. S25** Semi-quantitative analysis of intracellular Fluo-4/Ca²⁺ fluorescence intensity in PASMCs within the hypoxia-injured co-culture system treated with PSS31, NPF, HP, or HPF. Data were presented as mean±SD, n=3. ^*^*P*<0.05, ^**^*P*<0.01. Statistical significance was calculated *via* one-way ANOVA with Tukey’s test.

**Fig. S26** Distribution of HPF in the major organs of HPH rats. (a) Semiquantitative statistic results of fluorescence intensity in main organs from HPH rats after intravenous injection of HPF. (b) Semiquantitative statistic results of fluorescence intensity in main organs from HPH rats or normoxic rats after intravenous injection of HPF or HP. Data were presented as mean±SD, n=3. ^*^*P*<0.05, ^**^*P*<0.01. Statistical significance was calculated *via* one-way ANOVA with Tukey’s test.

**Fig. S27** Semiquantitative statistic results of fluorescence intensity of HPF and HP in lungs in HPH rats or normoxic rats. Data were presented as mean±SD, n=3. ^*^*P*<0.05, ^**^*P*<0.01. Statistical significance was calculated *via* one-way ANOVA with Tukey’s test.

**Fig. S28** Immunofluorescence images showing ROS level in lung tissues of HPH rats or normoxic rats. (red: DHE; blue: DAPI).

**Fig. S29** Echocardiographic images of HPH rats or normoxic rats.

**Fig. S30** Co-localization analysis of macrophages (M1/M2 type) and vascular smooth muscle cells in lung tissues of HPH rats or normoxic rats (green: CD86; red: α-SMA; purple: CD206; blue: DAPI).

**Fig. S31** TNF-α (a), IL-6 (b) and IL-1β (c) levels in lung tissues of HPH rats or normoxic rats. Data were presented as mean±SD, n=3. ^*^*P*<0.05, ^**^*P*<0.01. Statistical significance was calculated *via* one-way ANOVA with Tukey’s test.

**Fig. S32** Quantitative analysis of the expression of TRPC6, Kv1.5, and cyclin-D1 in lung tissues of HPH rats or normoxic rats. Data were presented as mean±SD, n=3. ^*^*P*<0.05, ^**^*P*<0.01. Statistical significance was calculated *via* one-way ANOVA with Tukey’s test.

**Fig. S33** Safety evaluation of HPF *in vivo* following the two-week treatment in normoxic rats. (a) H&E staining images of main organ tissues in normoxic rats. (b-e) Effects of HPF on the activity of serum ALT, AST, BUN, and CREA in normoxic rats. (f-i) Effect of HPF on prothrombin time, activated partial thromboplastin time, thrombin time, and fibrinogen in normoxic rats. Data were presented as mean±SD, n=5.
